# Systematic Evaluation of State Policy Interventions Targeting the US Opioid Epidemic, 2007-2018

**DOI:** 10.1001/jamanetworkopen.2020.36687

**Published:** 2021-02-12

**Authors:** Byungkyu Lee, Wanying Zhao, Kai-Cheng Yang, Yong-Yeol Ahn, Brea L. Perry

**Affiliations:** 1 Department of Sociology, Indiana University-Bloomington, Bloomington; 2 Luddy School of Informatics, Computing, and Engineering, Indiana University-Bloomington, Bloomington; 3 Network Science Institute, Department of Sociology, Indiana University-Bloomington, Bloomington

## Abstract

**Importance:**

In response to the increase in opioid overdose deaths in the United States, many states recently have implemented supply-controlling and harm-reduction policy measures. To date, an updated policy evaluation that considers the full policy landscape has not been conducted.

**Objective:**

To evaluate 6 US state-level drug policies to ascertain whether they are associated with a reduction in indicators of prescription opioid abuse, the prevalence of opioid use disorder and overdose, the prescription of medication-assisted treatment (MAT), and drug overdose deaths.

**Design, Setting, and Participants:**

This cross-sectional study used drug overdose mortality data from 50 states obtained from the National Vital Statistics System and claims data from 23 million commercially insured patients in the US between 2007 and 2018. Difference-in-differences analysis using panel matching was conducted to evaluate the prevalence of indicators of prescription opioid abuse, opioid use disorder and overdose diagnosis, the prescription of MAT, and drug overdose deaths before and after implementation of 6 state-level policies targeting the opioid epidemic. A random-effects meta-analysis model was used to summarize associations over time for each policy and outcome pair. The data analysis was conducted July 12, 2020.

**Exposures:**

State-level drug policy changes to address the increase of opioid-related overdose deaths included prescription drug monitoring program (PDMP) access, mandatory PDMPs, pain clinic laws, prescription limit laws, naloxone access laws, and Good Samaritan laws.

**Main Outcomes and Measures:**

The outcomes of interests were quarterly state-level mortality from drug overdoses, known indicators for prescription opioid abuse and doctor shopping, MAT, and prevalence of drug overdose and opioid use disorder.

**Results:**

This cross-sectional study of drug overdose mortality data and insurance claims data from 23 million commercially insured patients (12 582 378 female patients [55.1%]; mean [SD] age, 45.9 [19.9] years) in the US between 2007 and 2018 found that mandatory PDMPs were associated with decreases in the proportion of patients taking opioids (−0.729%; 95% CI, −1.011% to −0.447%), with overlapping opioid claims (−0.027%; 95% CI, −0.038% to −0.017%), with daily morphine milligram equivalent greater than 90 (−0.095%; 95% CI, −0.150% to −0.041%), and who engaged in drug seeking (−0.002%; 95% CI, −0.003% to −0.001%). The proportion of patients receiving MAT increased after the enactment of mandatory PDMPs (0.015%; 95% CI, 0.002% to 0.028%), pain clinic laws (0.013%, 95% CI, 0.005%-0.021%), and prescription limit laws (0.034%, 95% CI, 0.020% to 0.049%). Mandatory PDMPs were associated with a decrease in the number of overdose deaths due to natural opioids (−518.5 [95% CI, −728.5 to −308.5] per 300 million people) and methadone (−122.7 [95% CI, −207.5 to −37.8] per 300 million people). Prescription drug monitoring program access policies showed similar results, although these policies were also associated with increases in overdose deaths due to synthetic opioids (380.3 [95% CI, 149.6-610.8] per 300 million people) and cocaine (103.7 [95% CI, 28.0-179.5] per 300 million people). Except for the negative association between prescription limit laws and synthetic opioid deaths (−723.9 [95% CI, −1419.7 to −28.1] per 300 million people), other policies were associated with increasing overdose deaths, especially those attributed to non–prescription opioids such as synthetic opioids and heroin. This includes a positive association between naloxone access laws and the number of deaths attributed to synthetic opioids (1338.2 [95% CI, 662.5 to 2014.0] per 300 million people).

**Conclusions and Relevance:**

Although this study found that existing state policies were associated with reduced misuse of prescription opioids, they may have the unintended consequence of motivating those with opioid use disorders to access the illicit drug market, potentially increasing overdose mortality. This finding suggests that there is no easy policy solution to reverse the epidemic of opioid dependence and mortality in the US.

## Key Points

**Question:**

Are US state drug policies associated with variation in opioid misuse, opioid use disorder, and drug overdose mortality?

**Findings:**

In this cross-sectional study of state-level drug overdose mortality data and claims data from 23 million commercially insured patients in the US between 2007 and 2018, state policies were associated with a reduction in known indicators of prescription opioid misuse as well as deaths from prescription opioid overdose and increases in diagnosis of opioid use disorder, overdose, and drug overdose mortality from illicit drugs.

**Meaning:**

Although existing state-level drug policies have been associated with a decrease in the misuse of prescription opioids, these policies may have had the unintended consequence of motivating those with opioid use disorders to switch to alternative illicit substances, inducing higher overdose mortality.

## Teaser

This cross-sectional study assesses state drug policies implemented between 2007 and 2018 to ascertain whether they are associated with variations in opioid misuse, opioid use disorder, and drug overdose mortality.

## Introduction

The current opioid epidemic in the US has its historical roots in the movement during the 1990s to address undertreated chronic pain. In response, opioid-producing pharmaceutical companies engaged in aggressive marketing and prescribers overcorrected, relying on powerful opioid analgesics to treat acute and minor pain in addition to chronic and severe pain. Subsequently, the widespread use of opioid analgesic agents created demand for long-term and non-medical use of prescription and illicit opioids.^1,2^ To address the growing opioid epidemic, policy makers have focused largely on controlling the prescription and use of opioid analgesics through the implementation of supply-side drug policies. These include prescription drug monitoring programs (PDMPs), pain clinic laws, and prescription limit laws to reduce inappropriate prescribing behavior. In tandem, policy measures to reduce harms and barriers associated with treating and reporting drug overdose, including naloxone access laws and Good Samaritan laws, have been introduced.

Previous studies have provided mixed evidence on the impact that these state policies have had on opioid misuse, nonfatal overdose, and opioid mortality. For example, some research indicates that access to a PDMP, which allows prescribers to review patients’ prescription histories, substantially reduces prescription of opioids by 6 percentage points and oxycodone distribution by 8 percentage points.^3,4^ In contrast, other studies have found that PDMP access is ineffective^5,6,7^ or is only effective if a review of patient records is mandatory.^8,9^ Other types of policies such as naloxone access and pain clinic laws have reported contradictory evidence.^10,11,12^ For example, naloxone access laws were estimated by 1 study to reduce opioid-related fatal overdose by 0.387 per 100 000 people in 3 or more years after adoption,^10^ whereas another study reported that there were no significant changes in mean opioid-related mortality but a 14% increase in the Midwest after the implementation of naloxone access laws.^11^

A likely explanation for the conflicting evidence on opioid policy outcomes is methodological limitations of existing work. First, discrepant findings may be attributable to differences in data coverage, such as variation in the states and time periods included in analyses.^13,14^ This sample selection issue makes it difficult to compare and synthesize existing evidence. Second, there is disagreement about how to operationalize and model the timing of policy implementation.^15^ Third, the most common modeling approach, the 2-way fixed-effects model in difference-in-differences analysis (ie, controlling for both state and period indicators) has an important limitation: likely violation of the parallel trends assumption.^16,17^ Because many states enacted new policies after 2013, it is urgent to conduct an updated assessment of the impact of state opioid policy using more recent data.

Herein, we present the most comprehensive study to date on state policies that target the US opioid epidemic, focusing on the consequences of policies for both prescription opioid misuse and overdose mortality. Are state drug policies significantly associated with variations in opioid misuse, opioid use disorder, and drug overdose mortality? To answer this question, we use panel matching to implement a rigorous difference-in-differences approach in conjunction with extensive data coverage that includes observations through 2018 (2007–2018; across 50 states). We also assemble and refine the policy timing data across the 6 most widely studied policies; to our knowledge, these data have never before been investigated in tandem.

## Methods

This analysis draws on medical and pharmacy claims data from the Optum Clinformatics Data Mart Database (2007-2018) and a publicly available mortality data set from the National Center for Health Statistics (NCHS). The Optum database is a large deidentified database from a national private insurance provider^18,19,20^ that includes medical and prescription claims for the full population of patients ever prescribed any controlled substance between 2007 and 2018 (approximately 23 million). eAppendix 1 and eTable 1 in the Supplement include a description of the Optum database and the population coverage by state. Excluded from the study were patients with cancer and those receiving palliative care because they are expected to be outliers with respect to (medically necessary) opioid consumption. We measured all indicators at the patient level and aggregated them to the level of state of residence. We obtained state-level overdose mortality data from the NCHS Multiple Cause of Death file from 1999 to 2018. In addition, we obtained data on several confounders (ie, the proportion of female individuals; those aged <40 years, 40-60 years, and >60 years; White, Black, Asian, and Hispanic individuals, and individuals of other races [American Indian, Pacific Islander, and multiple racial categories except for Hispanic]; those who were unemployed; those living below the poverty line; the state population; and state implementation of Medicaid expansion) from current population surveys. We aggregated individual-level data to quarterly state-level data using the provided survey weights. Herein we report results across quarterly units because we find that these units generate more precise estimates. This study was approved by the institutional review board at Indiana University, and the requirement for informed consent was waived because deidentified data were used. We followed the Strengthening the Reporting of Observational Studies in Epidemiology (STROBE) reporting guideline.

### Prescription Opioid Indicators

We used an extensive set of prescription opioid outcomes. First, we measured the proportion of patients who received any opioid during a given period (ie, quarter). Second, using the subset of patients who received any opioid between 2007 and 2018, we computed 5 additional measures. The *International Classification of Diseases, Ninth Revision *(*ICD-9*) and *International Statistical Classification of Diseases and Related Health Problems, Tenth Revision *(*ICD-10*) codes were used to identify opioid use disorder and overdose diagnosis (eTable 2 in the Supplement) and measure the proportion of patients with the disorder or overdose diagnosis. We also calculated the proportion of patients who received opioid doses higher than the maximum daily morphine milligram equivalent (MME) of 90, which was generated by enumerating daily MME (Strength per Unit × [Number of Units/Days Supply] × MME Conversion Factor)^21^ of all opioid prescriptions over time for each patient.

In addition, we measured the traditional doctor-shopping indicator (ie, the proportion of patients who visit ≥4 unique doctors and ≥4 unique pharmacies for opioids within 90 days)^22^ and the proportion of patients with overlapping opioid prescriptions. We also examined opioid treatment, which was defined as the proportion of patients who were prescribed any medication-assisted treatment (MAT) drug that includes buprenorphine, buprenorphine/naloxone, buprenorphine hydrochloride and naltrexone.^23^ We excluded methadone from the MAT drug list because it is often used as an opioid analgesic when prescribed by primary care physicians and other physicians for purposes other than substance use treatment, although results were similar when methadone was included in the list. Across all measures, we used the number of total enrollees, excluding those with cancer and those receiving palliative care in each state and year-quarter as denominators. eTable 3 in the Supplement details the basic characteristics of the analytic sample. eTable 4 in the Supplement includes summary statistics for the 6 indicators across patients with the Medicare low-income subsidy, Medicare beneficiaries, and patients without Medicare.

### Overdose Mortality

We used *ICD-10* codes to identify overdose mortality and to differentiate the cause of death. Deaths with drug overdose as the underlying cause were first identified using codes X40-X44 (unintentional), X60-X64 (suicide), X85 (homicide), and Y10-Y14 (undetermined intent). Of those codes, drug-related deaths were further identified based on codes T40.0-T40.6, including those for heroin (T40.1), natural and semisynthetic opioids (T40.2), methadone (T40.3), synthetic opioids excluding methadone (T40.4), cocaine (T40.5), and other unspecified drugs (T40.6). We included opioid and nonopioid overdose-inducing drugs because of the high incidence of polysubstance use among those with opioid use disorder.^24,25^ We repeated our query through the Centers for Disease Control and Prevention’s CDC Wonder online database across different causes of death (*ICD-10* codes T40.1-T40.6) between 1999 and 2018 except for 1 month, and then subtracted the total number of deaths excluding each month from the total number of deaths across 2007 to 2018 (a total of 144 queries) to identify the number of deaths each month. This method can was used to recover the monthly death counts (eTable 5 in the Supplement includes the annual counts across different causes of deaths).

### State Policies

We compiled a data set on 6 opioid-related policies with 2 broad objectives: to control the supply of prescription opioids or reduce harms and barriers to medical assistance for overdose. eTables 6 and 7 in the Supplement show the exact year and month of all policy implementation dates by state. The opioid-related policies include (1) PDMP access laws that provide access to the PDMP, an electronic database that tracks controlled substance prescriptions in a state; (2) mandatory PDMPs that require prescribers under certain circumstances to access the PDMP database prior to prescribing opioids; (3) prescription limit laws that impose limitations on the number of days that medical professionals dispense opioids for acute pain; (4) pain clinic laws that regulate the operation of pain clinics; (5) Good Samaritan laws that provide immunity or other legal protection for those who call for help during overdose events; and (6) naloxone access laws that provide civil or criminal immunity to licensed health care clinicians or lay responders for administration of opioid antagonists, such as naloxone hydrochloride, to reverse overdose. eAppendix 2 in the Supplement describes the information collection process for these state policies. Based on the dates of these policies, we defined *treatment indicators*, which were assigned a value of 1 if the law was active in a given quarter, otherwise a value of 0 was assigned. In addition, we created a separate indicator for state implementation of Medicaid expansion that we obtained from the Kaiser Family Foundation to control for its potential impact on the statistical inferences.

### Statistical Analysis

To ascertain whether state policy altered the prevalence of opioid abuse and misuse indicators, we used a difference-in-differences approach. A state was considered to be a treated case if the policy had been changed at a specified time*,* otherwise it was considered to be an untreated case. The goal was to compare the outcomes of interest for a state under the new policy regime at a specific time with the outcome under the old policy regime if the policy was not enacted at the same time. The key challenge was to find a suitable control case for the treated case. The difference-in-differences approach imputed the change of outcomes in the control case as a comparison case for the change of outcomes in the treatment case under the parallel trends assumption (ie, potential outcomes have a parallel time trend for the treatment and control groups). Namely, the difference of outcomes between the treated case and the control case before the policy change would stay the same after the change in the absence of treatment.

To mitigate potential violations of the parallel trends assumption, we used panel matching to construct a matched data set to make trends of pretreatment outcomes parallel across control and treatment cases in addition to other observed confounders (ie, the proportion of female individuals; individuals aged <40 years, 40-60 years, and >60 years; individuals of White, Black, Asian, and Hispanic race and those of other races [American Indian, Pacific Islander, and multiple racial categories except for Hispanic], unemployed individuals, those living below the poverty line, as well as the state population and state implementation of Medicaid expansion) through covariate-balancing propensity scores (CBPS). eAppendix 3 in the Supplement describes the application of panel matching.

We used the PanelMatch package in R, version 4.0.1 (R Foundation for Statistical Computing),^26^ to estimate the effect sizes of the temporal associations between policies and leading outcomes of interest for each quarter for 3 years (ie, up to 12 quarters). We used the metafor package in R^27^ for the random-effects meta-analysis model to summarize temporal associations for each policy and outcome pair (eAppendix 3 in the Supplement). We present uncertainty associated with effect sizes using 95% CIs to shift focus away from the null hypothesis testing toward showing the full range of effect sizes, we define an association as negative if the mean effect across all quarters is negative and a positive association if the mean effect is positive. In addition, associations are defined as significant if their 95% CIs do not overlap with zero. The data analysis was conducted July 12, 2020. All codes and data to replicate analyses are available at https://dataverse.harvard.edu/dataverse/bk.

## Results

**Figure 1.**
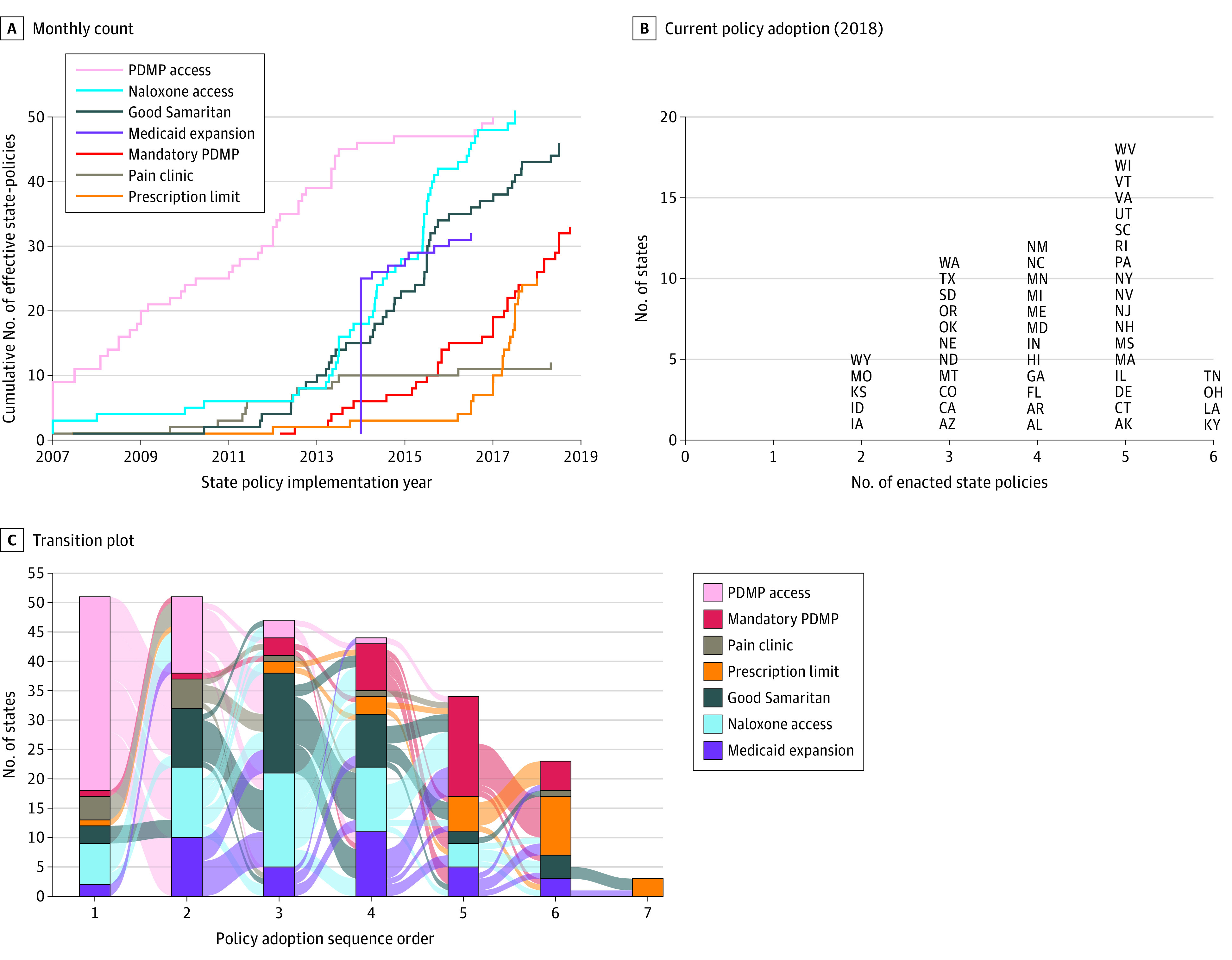
Implementation of State Policies From 2007 to 2018 Figure shows the cumulative number of opioid-related state policies implemented, with Medicaid expansion included as a control (A), the number of enacted state policies as of 2018 (B), and the transition rate of the state policies (C). PDMP indicates prescription drug monitoring program.

This cross-sectional study includes data on drug overdose mortality from the Optum Clinformatics Data Mart database and insurance claims data from the NCHS for 23 million commercially insured patients in 50 states between 2007 and 2018. Of these patients, 12 582 378 (55.1%) were women, and the mean (SD) age was 45.9 (19.9) years. Figure 1 depicts the cumulative count of opioid-related policies implemented by 50 states from 2007 to 2018. The monthly count of opioid policies started to increase substantially after 2012 to 2013. The implementation of Good Samaritan and naloxone access laws have been the largest contributor to the overall increase in policy adoption since 2013. In the first quarter of 2018, states adopted a mean of 4.1 policies, and 22 states adopted all but 1 policy. Access to PDMPs was implemented first by most states followed by the implementation of Good Samaritan and naloxone access laws, whereas mandatory PDMP and prescription limit laws were implemented in later periods. In 2014, Medicaid expansion laws were enacted in approximately half of the states, which may have biased the impact of other state drug policies. We accounted for this issue by controlling for an indicator of Medicaid expansion in the statistical models.

**Figure 2.**
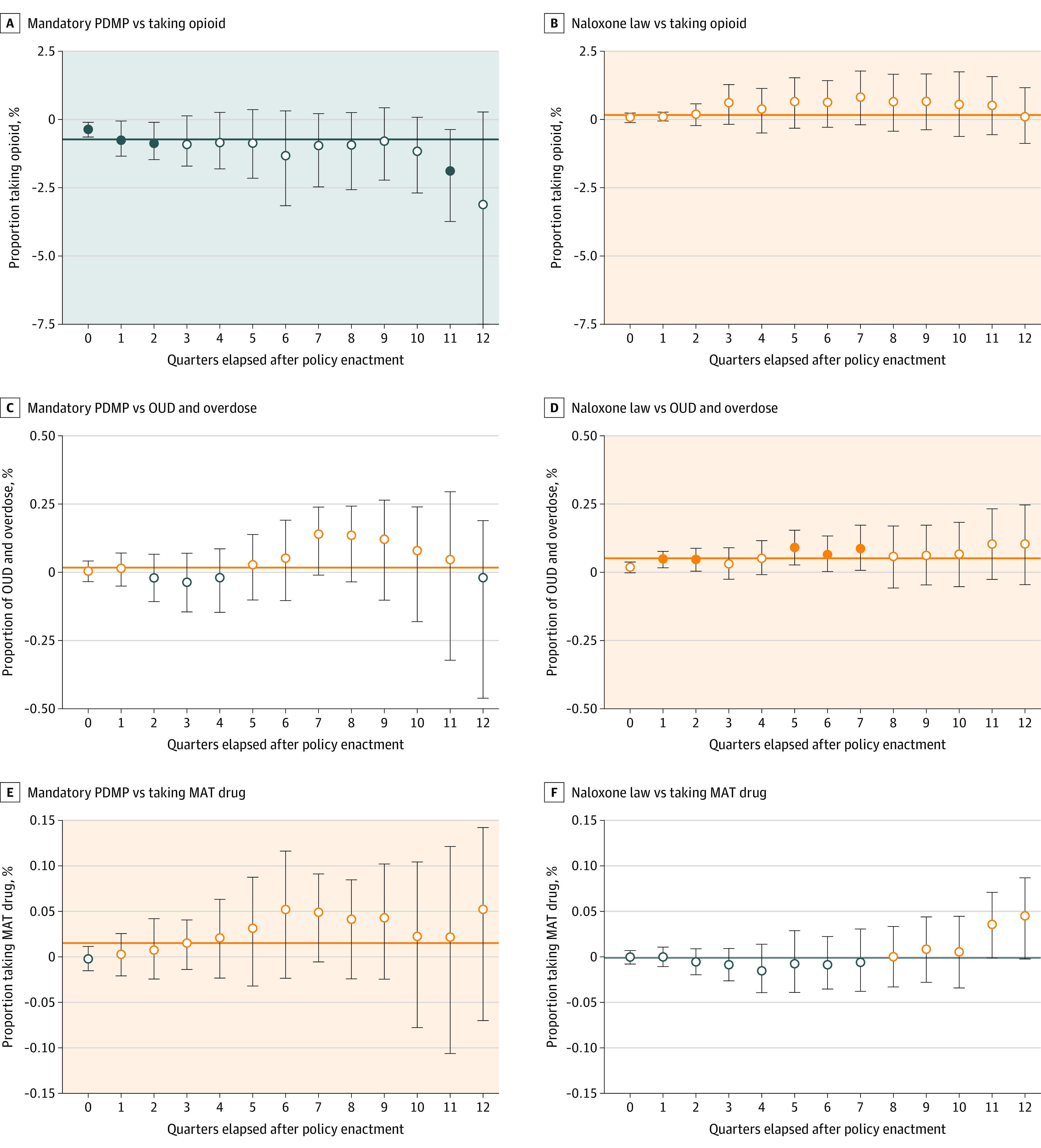
Association of Mandatory Prescription Drug Monitoring Program (PDMP) and Naloxone Law Implementation With Indicators of Prescription Opioid Misuse and Opioid Overdose in a Commercially Insured Population The error bars represent the 95% CIs, and the shaded backgrounds represent positive (orange) and negative (blue) associations. The solid horizontal line represents the estimated mean effect size across all quarterly effect sizes from random-effects meta-analysis models. Quarters are a period of three months. MAT indicates medication-assisted treatment; open circle, 95% CIs overlap with 0; OUD, opioid use disorder; solid circle, 95% CIs do not overlap with 0.

The rate of overdose deaths as well as the diagnosis of overdose and opioid use disorder increased as states implemented more policies (eFigure 4 in the Supplement), although indicators of opioid misuse and “doctor shopping” declined. For example, states without any of 6 policies exhibited a lower proportion of patients with overdose and opioid use disorder (0.14%) and a higher proportion of patients with a daily MME of 90 or higher (2.54%) vs states with all 6 policies (0.95% and 2.01%, respectively). Associations between mandatory PDMP and naloxone access law on 3 indicators from the commercially insured population are presented in Figure 2. eFigure 5 in the Supplement shows the consequences of implementation of all 6 policies for all 6 indicators. Figure 2 summarizes the temporal associations for each policy and outcome pair; the effect sizes with 95% CIs are presented with shaded panel backgrounds if the 95% CIs did not overlap with 0 effects (in eTable 8 in the Supplement).

First, supply-controlling policies were associated with reduction in 4 known prescription opioid misuse indicators: the proportion of patients who take opioids, have overlapping claims, receive higher opioid doses (daily MME ≥90), and visit multiple providers and pharmacies. For example, a mandatory PDMP was associated with a reduction of 0.729% (95% CI, −1.011% to −0.447%) in the proportion of patients taking opioids, those with overlapping opioid claims (−0.027%; 95% CI, −0.038% to −0.017%), those with a daily MME of 90 or higher (−0.095%; 95% CI, −0.150% to −0.041%), and in those who engaged in drug seeking behavior (−0.002%; 95% CI, −0.003% to −0.001%. In contrast, PDMP access was associated with a 0.17% (95% CI, 0.07%-0.26%) increase in the proportion of patients taking opioids and a 0.04% (95% CI, 0.007%-0.075%) increase in patients with a daily MME of 90 or higher. Second, the harm-reduction policies (ie, Good Samaritan laws and naloxone access laws) were associated with modest increases in the proportion of patients with overdose (0.014%; 95% CI, 0.002%-0.027%) and opioid use disorder (0.05%; 95% CI, 0.03%-0.07%). Third, the proportion of patients receiving MAT drugs increased following the implementation of supply-controlling policies, including mandatory PDMP (0.015%; 95% CI, 0.002%-0.028%), pain clinic laws (0.013%; 95% CI, 0.005%-0.021%), and prescription limit laws (0.034%; 95% CI, 0.020%-0.049%). Methadone as MAT produces similar results, including for pain clinic laws (0.024%; 95% CI, 0.009% to 0.039%) and prescription limit laws (0.014%, 95% CI, 0.003% to 0.025%). In general, the policy effect sizes became larger when estimating later outcomes, suggesting that the policy implementation does not produce immediate changes, but rather takes time to be effective (ie, a policy response lag).

**Figure 3.**
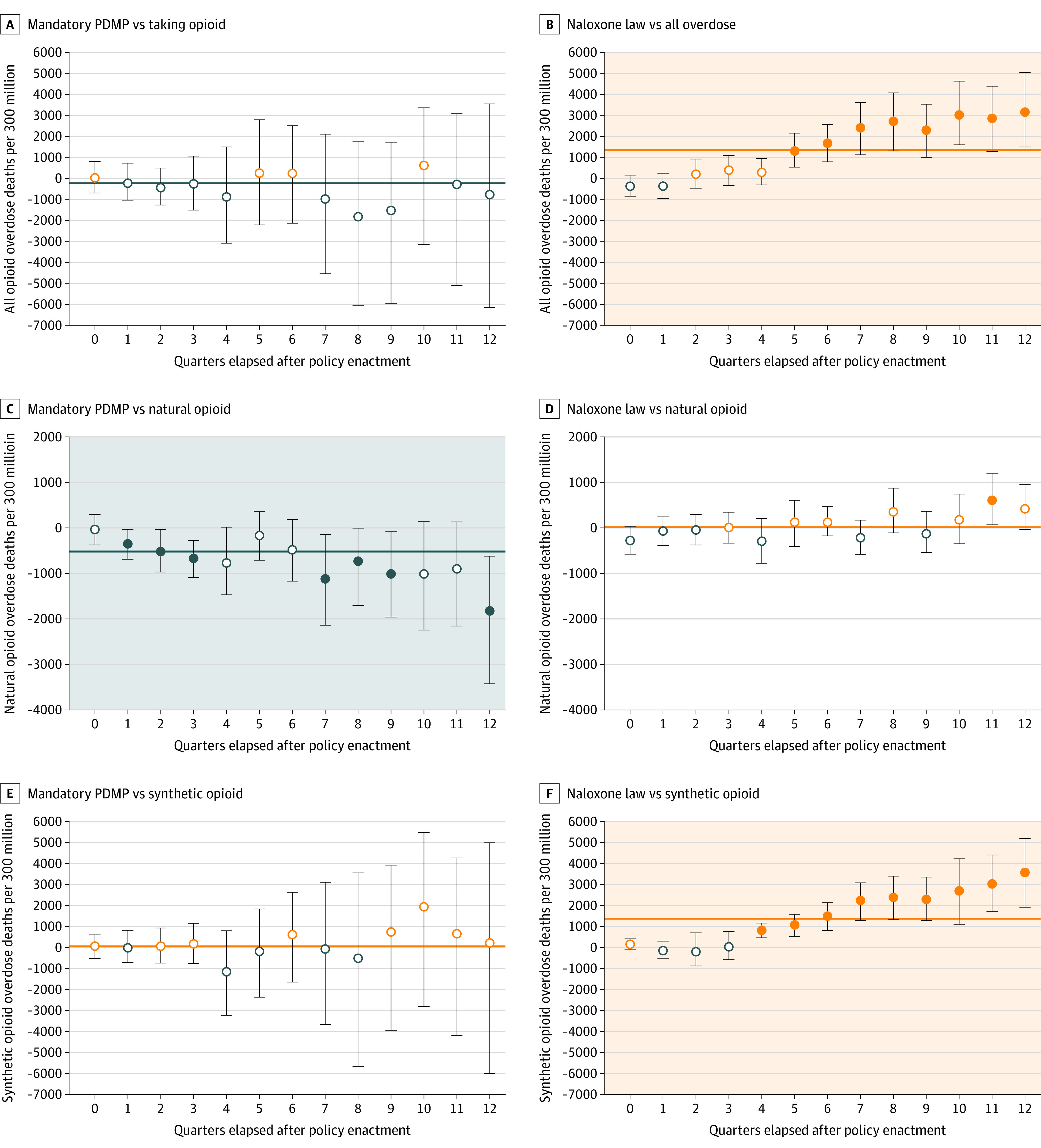
Association of Mandatory Prescription Drug Monitoring Program (PDMP) and Naloxone Law Enactment With Overall Drug Overdose Death and Death From Natural or Synthetic Opioid The error bars represent the 95% CIs, and the shaded backgrounds represent positive (orange) and negative (blue) associations. Open circles indicate that 95% CIs overlap with 0; solid circle, 95% CIs do not overlap with 0.

Figure 3 shows the rate of drug overdose mortality after enactment of mandatory PDMPs and naloxone access laws. eFigure 6 in the Supplement shows the rate of drug overdose mortality after enactment of all 6 policies, and eTable 9 in the Supplement shows the temporal associations for overall drug overdose deaths and deaths from specific opioids (eg, natural, synthetic, heroin). First, all overdose deaths increased following the implementation of naloxone access laws (1344.3 [95% CI, 627.1-2061.6] per 300 million people), especially deaths attributable to heroin (280.3 per 300 million people), synthetic opioids (1338.2 [95% CI, 662.5-2014.0] per 300 million people), and cocaine (557.6 [95% CI, 328.3-787.0] per 300 million people). Good Samaritan laws were also associated with increases in overall overdose deaths (403.7 [95% CI, 172.7-634.8] per 300 million people). Second, mandatory PDMPs were associated with a reduction of overdose deaths from natural opioids (−518.5 [95% CI, −728.5 to −308.5] per 300 million people) and methadone (−122.7 [95% CI, −207.5 to −37.8] per 300 million people), although the effect size was smaller than those of harm-reduction policies. Prescription drug monitoring program access policies showed similar results, although these policies were also associated with increases in overdose deaths from synthetic opioids (380.3 [95% CI, 149.6-610.8] per 300 million people) and cocaine (103.7 [95% CI, 28.0-179.5] per 300 million people). The implementation of pain clinic laws was associated with an increase in the number of overdose deaths from heroin (336.3 [95% CI, 79.5-593.0] per 300 million people) and cocaine (97.3 [95% CI, 23.9-170.8] per 300 million people). As an exception, having a prescription limit law was associated with a decrease in overdose deaths from synthetic opioids (−723.9 [95% CI, −1419.7 to −28.1] per 300 million people). In line with the results from analysis of 6 policies using medical claims data, we found a substantial policy response lag. All effect sizes from 0 to 12 quarters across 12 outcomes and 6 policies are presented in eTable 10 in the Supplement.

## Discussion

Recent trends in the US opioid epidemic present a paradox: opioid overdose mortality has continued to increase despite declines in opioid prescriptions since 2012.^28,29^ The opioid paradox may arise from the success—not failure—of state interventions to control opioid prescriptions. This finding is supported by a comprehensive assessment of multiple opioid policies on a range of outcomes, including opioid misuse and overdose mortality with extensive data coverage. We found that supply-controlling policies were associated with a reduction in the amount of prescription opioid misuse and the number of overdose deaths attributable to natural opioids as well as an increase in the number of patients receiving MAT drugs. In tandem, the significant increase of overdose deaths from synthetic opioids, heroin, and cocaine after the enactment of PDMP access, pain clinic laws, and naloxone access laws suggests that current drug policies may have the unintended consequence of motivating opioid users to switch to illicit drugs. An important implication of our findings is that there is no easy policy solution to reverse the epidemic of opioid dependence and mortality in the US. Hence, to resolve the opioid paradox, it is imperative to design policies to address the fundamental causes of overdose deaths (eg, lack of economic opportunity, persistent physical, and mental pain) and enhance treatment for drug dependence and overdose rather than focusing on opioid analgesic agents as the cause of harm.^2,30^

Prescription drug monitoring programs are the most widely studied policy responses to the opioid epidemic. Previous research on their impact indicates that providing access to PDMPs is not associated with significant improvement, but PDMPs have reduced prescription opioid misuse when accessing the databases was required for physicians.^8,22,31^ The present study found that mandatory PDMPs also reduced opioid misuse in a commercially insured population. In addition, we found that prescription limit laws and pain clinic laws were associated with a reduction in opioid abuse and an increase in the proportion of patients receiving MAT drugs. A study found that these laws as designed significantly reduce the length of initial prescription, although they also increase the likelihood of new (ie, first time) opioid use and the strength of initial prescription.^32^ In addition, pain clinic laws have been associated with modest decreases in opioid prescribing in Florida and Texas.^33,34^ The results of the present study are broadly consistent with those of other studies.

This extensive analysis may settle some of the contradictory findings in the literature and contributes to the previous research on opioid policy outcomes. Previous research on naloxone access and Good Samaritan laws has yielded inconsistent results. Namely, the enactment of naloxone access laws has been associated with substantial reductions in a fatal overdose but increased nonfatal overdoses.^10,12^ These results, however, have been contradicted by other studies that suggested that expansion of naloxone access laws leads to more opioid-related emergency department visits and thefts without any substantial reductions in opioid mortality.^11,35^ Likewise, previous research has suggested that Good Samaritan laws are not associated with heroin-related mortality, but substantially reduce mortality from other opioids.^12^ The situation is similar to studies examining the association between PDMPs and overdose mortality.^6,7,13,14^ Differences between our results and those of previous studies may be explained by our rigorous and extensive analytic approach, which uses panel matching for difference-in-differences analysis to mitigate the violation of the parallel trends assumption and the use of more recent data while simultaneously examining temporal associations. Given that most state policies were enacted after 2013, the present study used data through 2018 to provide the most up-to-date evidence on the opioid policy landscape.

We believe that our findings on the role of harm reduction policies in accelerating drug overdose deaths, especially those attributable to synthetic opioids and heroin, have important implications. Good Samaritan laws are designed to remove the threat of liability for people who call for emergency assistance in the event of a drug overdose. It is theoretically possible that provision of immunity may lead to greater reporting of overdose events in the absence of actual increases, although it is less likely to explain the increase of overdose deaths after the enactment of a Good Samaritan law. Naloxone laws provide civil immunity to licensed health care professionals or lay responders for opioid antagonist administration. Although expanded access to naloxone can reverse an opioid overdose and save lives, we found that naloxone access laws were associated with a substantial increase rather than a decrease in overdose deaths, especially deaths from illicit drugs. It is possible that the prospect of getting access to overdose-reversing treatment may instead induce moral hazards by encouraging people to use opioids and other drugs in riskier ways than they would have without the safety net of naloxone.^11^ Although naloxone access laws were estimated to increase naloxone dispensing,^36^ a recent study found that only 2135 of 138 108 high-risk patients (1.5%) in the US were prescribed naloxone in 2016.^37^ This finding suggests that policies designed to dramatically improve treatment for overdose are needed.

### Limitations

Several important limitations of this study are worth noting. First, a difference-in-differences approach through panel matching cannot account for spillover effects between states and between policies.^26^ Although we believe that considering multiple types of drug policies simultaneously is important, teasing out the association between a single policy and outcome from those of other policies is difficult because of correlated policy responses. Likewise, because prior research suggests that states’ adoption of Medicaid expansion has led to an increase in opioid overdose-related mortality^38^ but a decrease in opioid-related hospital use,^39^ we accounted for this factor by adjusting for Medicaid expansion in the modeling framework. However, identifying policy consequences on outcomes while controlling for the adoption of Medicaid expansion may produce underestimates or overestimates given the policy response lag and overlaps among different policy responses. Future research may consider, for example, sequence analysis or other clustering methods combined with the difference-in-differences approach to examine the impact of correlated policy responses.

Second, because we draw on data from a commercially insured population, these findings may not extend to other populations or to those individuals with insurance who pay cash for opioid prescriptions. Opioid misuse and overdose rates are higher among Medicare beneficiaries in our patient population with a low-income subsidy (eTable 4 in the Supplement), which suggests that this analysis may be missing a substantial proportion of the population at risk for opioid problems. Although the results on opioid misuse are consistent with those of an earlier report on mandatory PDMPs up to 2013 using a Medicare part D sample,^8^ future research is needed to ascertain whether the findings of the present analysis can be generalized to other patient populations. Third, there are potential limitations of using *ICD-10* codes to identify fatal overdose owing to inaccuracy and incompleteness of death classification.^40^ In addition, the transition of overdose coding from *ICD-9* to *ICD-10* may have contributed to the increase in overdose cases after October of 2015.^41^ Because systematic misreporting on fatal and nonfatal overdoses may induce bias in our estimates, it is crucial to examine and account for factors that affect the identification of overdose deaths in future work.

## Conclusions

The cause of opioid dependence is multifactorial, rooted in complex interactions between social, psychological, biological, and genetic factors.^42^ Heightened demand for diverted and illicit drugs might arise from limiting the supply of prescription opioids under certain conditions.^43^ These unintended consequences may occur if the fundamental causes of demand for opioids are not addressed and if the ability to reverse overdose is expanded without increasing treatment of opioid overdose. We believe that policy goals should be shifted from easy solutions (eg, dose reduction) to more difficult fundamental ones, focusing on improving social conditions that create demand for opioids and other illicit drugs.^2^

## Notes

Supplement. **eAppendix 1.** Introduction to Optum Database **eAppendix 2.** State Policies **eAppendix 3.** Application of Panel Matching Under the Parallel Trends Assumption **eTable 1. **Population Coverage of the Optum Database by State **eTable 2. **International Classification of Diseases (ICD), Ninth and Tenth Revision Codes to Identify Opioid Use Disorder (OUD) and Overdose Diagnosis (OD) **eTable 3.** Characteristics of the Study Samples From the Optum Database **eTable 4.** Summary Statistics for Six Indicators Across Medicare Beneficiary Status **eTable 5.** Overdose Death Counts Across Different Causes of Death **eTable 6.** Mandatory PDMP Implementation Dates **eTable 7.** State Policy Implementation Dates **eTable 8.** The Meta-Analysis of Quarterly Effects of State Policy Implementation on Indicators of Prescription Opioid Misuse and Opioid Overdose From a Commercially Insured Population **eTable 9.** The Meta-Analysis of Quarterly Effects of State Policy Implementation on Overdose Deaths **eFigure 1.** Illustration of Parallel Trends Assumption Under the Difference-in-Difference Framework: The Impact of Mandatory PDMPs on All Overdose Deaths **eFigure 2.** Pre-Post Trends for Six Outcomes From a Commercially Insured Population Across Six Policies **eFigure 3.** Pre-Post Trends for Different Types of Drug-Related Deaths Across Six Policies **eFigure 4.** Trends for Major Outcomes **eFigure 5.** The Association of State Policy Implementation With Indicators of Prescription Opioid Misuse and Opioid Overdose From a Commercially Insured Population **eFigure 6.** The Association of State Policy Implementation With Different Types of Drug Overdose Deaths **eTable 10.** Regression Tables for Effects of State Policies on All Outcomes With 95% Confidence Intervals **eReferences**
