# Error in the Supplement

**DOI:** 10.1001/jamanetworkopen.2021.3558

**Published:** 2021-03-10

In the Original Investigation titled “Systematic Evaluation of State Policy Interventions Targeting the US Opioid Epidemic, 2007-2018,”^1^ published February 12, 2021, there were errors in the data reported in eTable 4 in the Supplement. The Supplement has been corrected.
