# Supplementary material for: Systematic Evaluation of State Policy Interventions Targeting the US Opioid Epidemic, 2007-2018
Source: JAMA Netw Open. 2021 Feb 12;4(2):e2036687. doi: 10.1001/jamanetworkopen.2020.36687 (PMC7881356; doi:10.1001/jamanetworkopen.2020.36687)
Supplement: Supplement. — eAppendix 1. Introduction to Optum Database eAppendix 2. State Policies eAppendix 3. Application of Panel Matching Under the Parallel Trends Assumption eTable 1. Population Coverage of the Optum Database by State eTable 2. International Classification of Diseases (ICD), Ninth and Tenth Revision Codes to Identify Opioid Use Disorder (OUD) and Overdose Diagnosis (OD) eTable 3. Characteristics of the Study Samples From the Optum Database eTable 4. Summary Statistics for Six Indicators Across Medicare Beneficiary Status eTable 5. Overdose Death Counts Across Different Causes of Death eTable 6. Mandatory PDMP Implementation Dates eTable 7. State Policy Implementation Dates eTable 8. The Meta-Analysis of Quarterly Effects of State Policy Implementation on Indicators of Prescription Opioid Misuse and Opioid Overdose From a Commercially Insured Population eTable 9. The Meta-Analysis of Quarterly Effects of State Policy Implementation on Overdose Deaths eFigure 1. Illustration of Parallel Trends Assumption Under the Difference-in-Difference Framework: The Impact of Mandatory PDMPs on All Overdose Deaths eFigure 2. Pre-Post Trends for Six Outcomes From a Commercially Insured Population Across Six Policies eFigure 3. Pre-Post Trends for Different Types of Drug-Related Deaths Across Six Policies eFigure 4. Trends for Major Outcomes eFigure 5. The Association of State Policy Implementation With Indicators of Prescription Opioid Misuse and Opioid Overdose From a Commercially Insured Population eFigure 6. The Association of State Policy Implementation With Different Types of Drug Overdose Deaths eTable 10. Regression Tables for Effects of State Policies on All Outcomes With 95% Confidence Intervals eReferences [file jamanetwopen-e2036687-s001.pdf]

Supplemental Online Content

Lee B, Zhao W, Yang KC, Ahn YY, Perry BL. Systematic evaluation of state policy interventions targeting the US opioid epidemic, 2007-2018. *JAMA Netw Open*. 2021;4(2):e2036687. doi:10.1001/jamanetworkopen.2020.36687

eAppendix 1. Introduction to Optum Database
eAppendix 2. State Policies
eAppendix 3. Application of Panel Matching Under the Parallel Trends Assumption
eTable 1. Population Coverage of the Optum Database by State
eTable 2. International Classification of Diseases (ICD), Ninth and Tenth Revision Codes to Identify Opioid Use Disorder (OUD) and Overdose Diagnosis (OD)
eTable 3. Characteristics of the Study Samples From the Optum Database
eTable 4. Summary Statistics for Six Indicators Across Medicare Beneficiary Status
eTable 5. Overdose Death Counts Across Different Causes of Death
eTable 6. Mandatory PDMP Implementation Dates
eTable 7. State Policy Implementation Dates
eTable 8. The Meta-Analysis of Quarterly Effects of State Policy Implementation on Indicators of Prescription Opioid Misuse and Opioid Overdose From a Commercially Insured Population
eTable 9. The Meta-Analysis of Quarterly Effects of State Policy Implementation on Overdose Deaths
eFigure 1. Illustration of Parallel Trends Assumption Under the Difference-in-Difference Framework: The Impact of Mandatory PDMPs on All Overdose Deaths
eFigure 2. Pre-Post Trends for Six Outcomes From a Commercially Insured Population Across Six Policies
eFigure 3. Pre-Post Trends for Different Types of Drug-Related Deaths Across Six Policies
eFigure 4. Trends for Major Outcomes
eFigure 5. The Association of State Policy Implementation With Indicators of Prescription Opioid Misuse and Opioid Overdose From a Commercially Insured Population
eFigure 6. The Association of State Policy Implementation With Different Types of Drug Overdose Deaths
eTable 10. Regression Tables for Effects of State Policies on All Outcomes With 95% Confidence Intervals
eReferences

This supplemental material has been provided by the authors to give readers additional information about their work.

eAppendix A. Introduction to Optum Database

Our analysis draws on medical and pharmacy claims data from the Optum Clinformatics Data Mart Database (2007-2018) as well as a publicly available mortality dataset from the National Center for Health Statistics. The Optum database is a large deidentified database from a national private insurance provider[1–3] that includes medical and prescription claims for the full population of patients ever prescribed any controlled substance between 2007 and 2018 (approximately 23 million). This database covers about 20% of the entire commercially insured population in all 50 US States and Washington DC and 24% of the Medicare Advantage population. Patients in this dataset are of similar age and sex to the US commercially insured population more broadly (see eTable 1 for the state-level coverage). Using this database, previous studies reported that annual opioid use prevalence was 14% for commercial beneficiaries, 26% for aged Medicare beneficiaries, and 52% for disabled Medicare beneficiaries[4]. eTable 2 describes characteristics of our analytic sample drawn from the Optum database, and eTable 3 shows summary statistics for six outcome indicators used in this study across Medicare beneficiary status.

© 2021 Lee B et al. *JAMA Network Open.*

eAppendix B. State Policies

As a representative policy to control the opioid supply, governments have enacted *prescription drug monitoring programs* (PDMPs). We follow Horwitz and colleagues[5]' collection of *PDMP operational* dates (i.e., when the full PDMP program becomes available to any end user). With regard to the *mandatory PDMP* (i.e., that which requires prescribers under certain circumstances to access the PDMP prior to prescribing opioids), we combine and compare six sources, resolving discrepancies between them (see eTable 6). In addition to the PDMPs, states have implemented two other notable policies during the observation period. These are *prescription limit laws,* which impose limitations on the number of days that medical professionals prescribe or dispense opioids for acute pain, and *pill mill laws,* which regulate the operation of pain clinics. We obtained information on prescription limit laws from Davis *et al.*[6], and pill mill laws from the Prescription Drug Abuse Policy System (PDAPS). To reduce harms related to overdose events and treatment, states have enacted *good Samaritan laws* that provide immunities or other legal protections for those who call for help during overdose events. These laws aim to reduce the fear of overdose bystanders being arrested for drug-related crimes. In addition, *naloxone access laws* provide civil or criminal immunity to licensed healthcare providers or lay responders for administration of opioid antagonists such as naloxone hydrochloride to reverse overdose. We obtained data from PDAPS to identify the enactment dates of good Samaritan laws and naloxone access laws in each state. We state-level Medicaid expansion data from Kaiser Family Foundation (https://www.kff.org/health-reform/state-indicator/state-activity-around-expanding-medicaid-under-the-affordable-care-act to control for its potential influence on our statistical inference: last accessed as of May 10, 2020). eTable 7 presents detailed schedules of all seven policies.

© 2021 Lee B et al. *JAMA Network Open.*

eAppendix C. Application of Panel Matching Under the Parallel Trends Assumption

A difference-in-difference (DID) design has been widely used as a quasi-experimental design to identify the effects of state policy changes on opioid abuse and misuse indicators. Panel A in eFigure 1 shows a graphical representation of the core logic behind the DID approach explained above. The parallel trends assumption is a critical foundation of the DID approach. However, the assumption is often violated if the treated states face stronger pressure to enact the state policy due to the rapid increase of opioid mortality and other opioid misuse indicators, compared to control states without it. In this case, the policy timing is no longer considered exogenous to potential outcomes. To mitigate these likely violations, we use panel matching to construct a weighted data set that makes trends of pretreatment outcomes parallel across control and treatment units[7]. In contrast to synthetic control methods, the proposed method allows us to flexibly estimate the causal effects of multiple treated units simultaneously. For each treated state, we first construct a matched set by pruning control states that do not share the identical treatment history in two previous years (eight quarters) and retain control states that do not receive any treatment for *F* periods when we estimate the treatment effect on the leading outcomes at quarter *F* after three years (0 to 12 quarters). This matching procedure reduces extrapolation biases due to model misspecification[7,8].

We further adjust for observed confounders through covariate-balancing propensity scores (CBPS)[9] including three years of lagged outcomes as well as state-level variables (% Female, % Age 40-60, % Age 60+, % White, % Blacks, % Asian, % Hispanic, % unemployed, % living below the poverty line, and the number of state population). In addition, we account for state implementation of Medicaid expansion in balancing scores[10]. We illustrate the importance of the parallel-trends assumption in eFigure 1 using a case for examining the impact of the mandatory PDMP policy on all overdose deaths as an example. Panel B in eFigure 1 shows that states did mandate PDMP access in

© 2021 Lee B et al. *JAMA Network Open.*

the wake of experiencing linearly increasing trends of all overdose deaths, where states without mandatory PDMP policy experienced a much weaker slope of changes at the same time. It also confirms that our application of panel matching makes the pre-trend of the matched set approximate that of the treated states, which is also consistent across other outcomes and other policies (see eFigure 2 and 3 for all other results). Panel C shows that the violation of the parallel-trends assumption poses a challenge for the event study framework since the increase of all overdose deaths after the implementation of mandatory PDMP may be part of continuing trends in a linear increase of all overdose deaths. Panel D shows that the effect becomes much weaker from estimating panel matching models that fix the likely violation of the parallel-trends assumption.

Finally, the recent methodological literature suggests that the standard method for conducting difference-in-difference (DID) analysis may produce biased estimates. The most widely used method, two-way linear fixed effects regressions, is mathematically equivalent to the DID estimator in a simple case of two time periods and two groups. However, this equivalence does not hold when there are multiple periods and multiple groups[11,12] due to the inconsistent standard errors arising from serial correlation[13], biases in coefficients associated with temporal treatment heterogeneity [14], and negative weights[15]. To address the challenges arising from employing two-way fixed effects models for DID analysis, we compute a weighted difference-in-difference estimator to address methodological concerns that a standard method, two-way fixed effects models[12], for conducting DID analysis may produce biased estimates using *PanelMatch* package in **R**[7]. The basic intuition behind the weighted DID estimator is to use three distinctive sets — a within-time matched set, within-unit matched set, and adjustment set — for multi-period multi-group DID estimators, while discarding some group-time units that confound causal inference. For example, in 2012, Kentucky began to require that physicians check the PDMP database prior to providing a prescription. We first define the within-unit matched set that

© 2021 Lee B et al. *JAMA Network Open.*

contains the observations for Kentucky in previous time periods. We also define the within-time matched set as states that do not have a must-access PDMP in the same time period. Finally, we define the adjustment set, which contains the observations of the within-time matched set in the previous time periods. Then, the multi-period DID estimator is defined as the difference of outcomes between Kentucky at *t* and the within-unit matched set against the difference of outcomes between the within-time matched set and the adjustment set. Imai and Kim[12] show that the multi-period DID estimator is numerically equivalent to a weighted two-way fixed effects regression estimator and use the method of moment estimator to calculate the cluster-robust standard error.

To account for potential policy-response lags, we estimate the effects of policies on the leading outcomes at each quarter for three years (i.e., up to 12 quarters) based on the matched set described above. To facilitate the interpretation of a large number of over-time effects across six policies across 12 outcomes, we perform meta-analysis using random effects models on these quarterly effects for each policy and outcome combination. Specifically, we assume that each observed effect y_q at quarter q is the combination of the unknown true effect (θ_q) and the sampling error (ϵ_q), where $\theta_q = \mu + u_q$, $e_q \sim N(0, v_q^2)$ and $u_q \sim N(0, \tau^2)$. In this random effects model, we assume that differences in our matched sample sets across different quarters may introduce some random variability among the true effects in addition to the random sampling error. We use restricted maximum-likelihood estimation to estimate τ^2 given that the REML estimator is approximately unbiased and quite efficient using *metaphor* package in **R**[16]. Employing fixed effect models provides similar results.

eTable 1. Population Coverage of the Optum Database by State

State	Total Patients	Medicare	Medicare with LIS	Total Prescription	Total overdosed
TX	2,974,836	498,968	190,642	204,752,690	63,516
FL	2,244,856	565,100	177,166	169,658,088	48,082
CA	2,052,791	549,714	78,141	167,477,841	42,528
GA	1,365,300	306,706	99,518	129,817,404	24,499
OH	1,035,082	154,670	53,554	83,126,081	17,082
NC	967,921	286,752	100,767	95,806,530	20,699
AZ	821,364	217,098	41,635	65,188,193	21,064
CO	759,702	157,655	39,187	58,288,148	16,579
IL	759,119	144,476	22,390	52,492,928	10,261
MO	733,073	150,861	52,346	59,522,148	13,127
WI	684,096	169,845	78,899	58,872,354	14,626
MN	670,737	113,088	2,521	48,738,611	10,403
NY	639,038	212,635	90,416	54,692,299	12,263
VA	513,849	58,045	21,550	31,803,908	7,102
MD	474,291	12,830	9,399	29,672,838	7,479
IN	473,549	105,269	53,684	32,705,063	9,198
TN	463,086	77,850	20,743	33,266,520	12,746
WA	412,607	154,214	60,711	34,539,868	12,272
LA	397,750	5,681	510	26,526,136	7,252
NJ	373,596	85,279	26,097	25,108,023	6,647
PA	336,667	32,244	15,651	18,464,291	6,479
OK	331,840	51,883	12,343	24,758,523	8,329
SC	306,616	109,517	52,798	21,786,253	7,276
AL	303,590	133,946	44,169	29,449,604	11,033
UT	277,968	71,213	20,871	18,767,421	8,288
AR	236,341	49,362	26,008	16,046,455	5,124
MA	228,440	49,392	26,861	20,552,027	4,370
OR	224,648	67,308	35,973	17,802,553	5,552
IA	224,253	47,037	8,336	19,360,514	3,120
MI	215,134	6,300	606	9,318,761	3,135
CT	208,257	87,341	56,703	17,685,543	4,291
NE	193,339	25,338	5,743	15,344,763	2,287
MS	187,644	2,098	114	10,399,838	3,812
KY	184,430	9,633	1,834	11,139,635	3,880
KS	182,390	12,274	3,273	10,420,283	1,968
RI	180,052	43,155	14,399	19,905,905	4,158
NV	149,366	40,597	6,103	7,661,397	4,993
NM	125,390	25,798	18,433	8,518,540	3,358
ID	72,831	26,002	5,460	5,718,673	2,206
DC	50,377	6,317	5,858	3,170,202	939
WV	48,709	1,708	94	2,650,876	1,044
NH	47,636	11,131	1,767	2,965,460	1,166
ND	36,044	6,328	100	1,927,162	569
ME	31,912	12,969	2,963	2,356,420	987
HI	29,658	25,066	15,592	4,674,164	1,184

© 2021 Lee B et al. *JAMA Network Open.*

DE	26,980	2,695	1,784	1,637,072	514
SD	25,053	6,726	76	1,614,492	318
WY	22,617	2,267	388	1,246,725	342
MT	20,365	2,888	320	1,198,649	282
VT	12,069	7,378	1,503	1,119,626	346
AK	8,169	185	9	322,679	151

© 2021 Lee B et al. *JAMA Network Open.*

eTable 2. International Classification of Diseases (ICD), Ninth and Tenth Revision Codes to Identify Opioid Use Disorder (OUD) and Overdose Diagnosis (OD)

	ICD9	ICD10
Opioid use disorder (OUD)	304.00, 304.01, 304.02, 304.70, 304.71, 304.72, 305.50, 305.51, 305.52	F11 series except F11.21
Overdose diagnosis (OD)	965.00, 965.01, 965.02; 965.09, 970.1, E850.0, E850.1, E850.2, E935.0, E935.1, E935.2, E940.1	T40 series

© 2021 Lee B et al. *JAMA Network Open.*

eTable 3. Characteristics of the Study Samples From the Optum Database

	Full Sample (N=22,857,346)		Analytic Sample (N=13,493,867)		Cancer Patient (N=9,363,479)	
	Mean / %	SD	Mean / %	SD	Mean / %	SD
Age	45.931	19.924	40.401	19.32	53.899	17.988
Female (%)	0.551		0.508		0.611	
Low Income Subsidy in Medicare program (%)	0.049		0.043		0.058	
Enrolled in Medicare Part D program (%)	0.204		0.138		0.3	
Overdose + OUD patients (%)	0.021		0.018		0.025	
Number of prescriptions	78.315	126.723	47.679	85.713	122.465	159.097
Number of opioid prescriptions	5.115	15.169	3.671	11.414	7.195	19.147
Cancer Patients (%)	0.407		0		0.994	0.078
Palliative Care Patients (%)	0.01		0		0.025	0.156

Note. The full sample includes the complete sample of patients ever prescribed any controlled substance between 2007 and 2018 (approximately 23 million patients). The analytic sample excludes cancer patients and palliative care patients.

eTable 4. Summary Statistics for Six Indicators Across Medicare Beneficiary Status

	Non-medicare	Medicare	Medicare with LIS	All
Number of patient-quarters	265,760,865	55,865,425	13,661,567	335,287,857
Mean Age	39.46	72.79	68.55	46.20
% Female	52	59	66	54
Number of prescriptions	879,753,467	451,065,934	192,956,349	1,523,775,750
Number of opioid prescriptions	57,809,195	27,097,688	14,480,658	99,387,541
% MAT drug prescription	0.20	0.12	0.42	0.20
% OUD + overdosed patients	0.22	0.48	1.37	0.31
% taking opioids	11.09	20.69	36.16	13.71
% high MME	1.69	3.65	7.94	2.27
% overlapping claim	0.23	0.68	1.51	0.36
% 4+4+90	0.0065	0.0063	0.028	0.0074

Note. This table is based on the analytic sample excluding cancer patients and palliative care patients (N=13,493,867) across all 47 quarters from 2007 to 2018 Q3.

eTable 5. Overdose Death Counts Across Different Causes of Death

We could identify the unsuppressed monthly death count across 50 states using the public CDC wonder database by identifying the total death count except for each month, which is subtracted from the total death count. For example, to get the number of deaths for 2018 December, we first make a query to retrieve the total number of deaths due to T40.1 from 1999 January to 2018 December (A), and then make another query to retrieve the total number of deaths due to T40.1 from 1999 January to 2018 November (B), and subtract B from A (i.e., A-B). The suppressed column shows the number of deaths after we suppress the death below 10 following the CDC's privacy protection rule.

ICD 10 code	Total Number of Deaths	Total Number of Deaths (suppressed)	Difference	Suppression rate (%)
T40.1	115,568	110,596	4,972	4.3
T40.2	175,004	169,900	5,104	2.9
T40.3	70,214	63,078	7,136	10.2
T40.4	124,486	116,587	7,899	6.3
T40.5	125,481	119,983	5,498	4.4
T40.6	54,688	46,532	8,156	14.9
All causes	695,607	694,637	970	0.1

© 2021 Lee B et al. *JAMA Network Open.*

eTable 6. Mandatory PDMP Implementation Dates

This table presents a list of different dates for mandatory PDMP enactment reported by previous studies, and the final date we selected based on our own research on conflicted cases.

State	PDAPS	Pauly et al[17]	PEW[18]	Buchmueller and Carey[19]	Sacks et al[20]	Mallatt[21]	Meinhofer[22]	Final
AL						2017-3		
AK					2017-7	2017-7		2017-07
AZ					2017-10	2018-3		2018-03
AR					2017-1	2017-8		2017-08
CA	2017-01				2018-4	2018-10		2018-10
CO						2018-5		
CT	2015-10		2015-10		2015-10	2015-10		2015-10
DE				2012-h1	2012-3	2012-3	2012-03	2012-03
DC								
FL						2018-7		2018-07
GA		2014-07				2018-7		2018-07
HI						2018-6		2018-06
ID								
IL					2018-1	2018-1		2018-01
IN	2014-07	2014-07			2014-7	2019-1		2019-01
IA								
KS								
KY	2012-07	2012-07	2012-07	2012-h2	2012-7	2012-7	2012-07	2012-07
LA	2014-08			2008-h1	2008-1	2014-8	2014-08	2014-08
ME	2017-01					2017-1		2017-01
MD					2018-7	2018-7		2018-07
MA	2014-07	2014-07	2016-01		2014-7	2013-6		2016-10
MI						2018-1		2018-01
MN								
MS						2018-3		2018-03
MO								
MT								
NE								
NV	2015-10		2015-10	2007-h2	2007-10	2007-10	2007-10	2015-10
NH	2016-01				2016-1	2017-1		2016-01
NJ	2015-11		2015-11		2015-11	2015-7		2015-11
NM	2012-09	2012-09	2012-11	2012-h2	2012-9	2012-10	2017-01	2017-01
NY	2013-08	2013-08	2013-08	2013-h2	2013-8	2013-9	2013-08	2013-08
NC								
ND								
OH	2015-12		2015-04	2012-h1	2012-3	2011-11	2011-10	2015-04
OK	2015-11		2015-11	2011-h1	2011-3	2010-11	2010-11	2015-11
OR								
PA	2015-06		2015-06		2017-1	2017-1		2017-01
RI	2016-06		2015-03		2016-6	2014-7		2015-03
SC					2017-5	2016-4		2017-05
SD						2017-7		
TN	2013-04	2013-07	2013-04	2013-h2	2013-7	2013-1	2013-04	2013-04
TX					2019-9	2019-9		2019-09
UT					2017-5			2017-05
VT	2015-05	2013-11			2015-5	2013-11		2013-11
VA	2015-07				2015-7	2015-7		2015-07

© 2021 Lee B et al. *JAMA Network Open.*

WA								
WV	2012-06	2012-06	2013-05	2012-h2	2012-6	2012-6	2012-06	2013-05
WI	2017-04					2017-4		2017-04
WY								

Note. PDAPS: http://pdaps.org/ Last accessed on 29, October, 2018. See the following source for each conflict:
AL: no mandatory PDMP, see http://www.alabamapublichealth.gov/PDMP/faq.html (as of 06/18/19)
AZ: Since 2017-10 is the date for mandatory reporting, we chose 2018-04 for mandatory access. See https://pharmacypmp.az.gov/ (as of 06/18/19)
AR: See https://www.healthy.arkansas.gov/images/uploads/pdf/Official_PMP_Quarterly_Report_1st_Quarter.pdf
GA: See https://dph.georgia.gov/pdmp
IN: See http://www.ciproms.com/2018/12/new-requirements-for-indianas-prescription-drug-monitoring-program-inspect-begin-january-1/
LA: https://www.lsbme.la.gov/sites/default/files/documents/In%20The%20News%20Items/Lammico%20Schedule%20II%20Drugs%2010%206%202014.pdf
MA: http://www.eprescribing.org/tag/massachusetts-state-mandate/Effective
NV: https://nabp.pharmacy/wp-content/uploads/2016/06/NV102016.pdf
NH: http://www.pdmpassist.org/content/new-or-amended-pdmp-legislationregulations
NJ: https://www.njconsumeraffairs.gov/Adoptions/dirado_11072016.pdf
NM: http://www.pdmpassist.org/content/new-or-amended-pdmp-legislationregulations
OH: https://www.ohiopmp.gov/Documents/General/PHARMACIES_PRESCRIBERS/Requirements%20Prior%20to%20Prescribing%20-%20Opioids%20and%20Benzodiazepines.pdf
OK: https://www.insurancejournal.com/news/southcentral/2015/04/06/363125.htm
PA: https://www.mcneeslaw.com/changes-pennsylvania-drug-monitoring-program-january-1-2017-compliance-prescribing-physicians/
RI: http://health.ri.gov/publications/plans/2015PrescriptionDrugMonitoringProgramEnforcementPlan.pdf
SC:https://qioprogram.org/sites/default/files/editors/141/SouthCarolina_PDMP_withDisclaimer_CF_FINAL_508.pdf
TN: http://www.pdmpassist.org/pdf/Mandatory_conditions.pdf
VT: https://legislature.vermont.gov/statutes/fullchapter/18/084A
WV: https://wvbom.wv.gov/download_resource.asp?id=269

© 2021 Lee B et al. *JAMA Network Open.*

eTable 7. State Policy Implementation Dates

This table presents implementation dates for six different policies that control the supply-side of opioid consumption or enhance access to treatment, as well as the implementation date for Medicaid expansion. We have collected dates of policy adoption from the following sources: Horwitz and collegues[5]' Table 1, Davis and collegues[6]' Table 2, PDAPS for Naloxone Law, PDAPS for Good Samaritan Law, PDAPS for Pain Clinic Law as of 2018/10/29. PDAPS, Kaiser Family Foundation for Medicaid Expansion. Also see eTable 6 for mandatory PDMP dates.

state	Law authorizing access to PDMP	Law requiring PDMP	Prescription Limit Law	Pill Mill Law	Good Samaritan Law	Naloxone Access Law	Medicaid Expansion
AK	2012m1	2017m7	2017m7		2014m10	2016m3	2015m9
AL	2006m4			2013m5	2015m6	2015m6	
AR	2013m5	2017m8			2015m7	2015m7	2014m1
AZ	2008m12	2018m3		2018m4	2018m4	2016m8	2014m1
CA	2009m9	2018m10			2013m1	2008m1	2014m1
CO	2008m2				2012m5	2013m5	2014m1
CT	2008m7	2015m10	2016m7		2011m10	2003m10	2014m1
DC	2016m10				2013m3	2013m3	2014m1
DE	2012m8	2012m3	2017m4		2013m8	2014m8	2014m1
FL	2011m10	2018m7		2010m10	2012m10	2015m6	
GA	2013m5	2018m7		2013m7	2014m4	2014m4	
HI	2012m2	2018m6	2016m7		2015m7	2016m6	2014m1
IA	2009m3				2018m7	2016m5	2014m1
ID	2008m4				2018m7	2015m7	2020m1
IL	2009m12	2018m1	2012m1		2012m6	2010m1	2014m1
IN	2007m7	2019m1	2017m7		2016m7	2015m4	2015m2
KS	2011m4					2017m7	
KY	2001m7	2012m7	2017m6	2012m7	2015m3	2013m6	2014m1
LA	2009m1	2014m8	2017m8	2006m1	2014m8	2015m8	2016m7
MA	2011m1	2016m10	2016m3		2012m8	2012m8	2014m1
MD	2013m12	2018m7	2017m5		2014m10	2013m10	2014m1
ME	2005m1	2017m1	2017m1			2014m4	2019m1
MI	2003m1	2018m1			2017m1	2014m10	2014m4
MN	2010m4		2017m7		2014m7	2014m5	2014m1
MO					2017m8	2016m8	
MS	2008m7	2018m3		2011m4	2015m7	2015m7	
MT	2012m10				2017m5	2017m5	2016m1
NC	2007m7		2018m1		2013m4	2013m4	
ND	2008m10				2015m8	2015m8	2014m1

© 2021 Lee B et al. *JAMA Network Open.*

NE	2017m1				2017m8	2015m5	
NH	2014m10	2016m1	2017m1		2015m9	2015m6	2014m8
NJ	2012m1	2015m11	2017m5		2013m5	2013m7	2014m1
NM	2005m8	2017m1			2007m6	2001m4	2014m1
NV	2011m2	2015m10	2017m6		2015m10	2015m10	2014m1
NY	2013m6	2013m8	2016m7		2011m9	2006m4	2014m1
OH	2006m10	2015m4	2017m8	2011m5	2016m9	2014m3	2014m1
OK	2006m7	2015m11				2013m11	
OR	2011m9				2016m1	2013m6	2014m1
PA	2016m8	2017m1	2017m1		2014m12	2014m12	2015m1
RI	2012m9	2015m3	2017m3		2012m6	2012m6	2014m1
SC	2008m2	2017m5	2007m6		2017m6	2015m6	
SD	2012m3				2017m7	2016m7	
TN	2010m1	2013m4	2013m10	2011m5	2015m7	2014m7	
TX	2012m8	2019m9		2009m9		2015m9	
UT	2006m1	2017m5	2017m3		2014m3	2014m5	2020m1
VA	2006m6	2015m7	2017m3		2015m7	2013m7	2019m1
VT	2009m1	2013m11	2017m7		2013m6	2013m7	2014m1
WA	2012m1				2010m6	2010m6	2014m1
WI	2013m6	2017m4		2016m3	2014m4	2014m4	
WV	2013m5	2013m5		2012m6	2015m6	2015m5	2014m1
WY	2013m7					2017m7	

© 2021 Lee B et al. *JAMA Network Open.*

eTable 8. The Meta-Analysis of Quarterly Effects of State Policy Implementation on Indicators of Prescription Opioid Misuse and Opioid Overdose From a Commercially Insured Population

	PDMP access	mandatory PDMP	Pill Mill Law
P(taking opioid)	0.167 [0.074,0.26]	-0.729 [-1.011,-0.447]	-0.444 [-0.618,-0.27]
P(overlapping claims)	-0.004 [-0.014,0.006]	-0.027 [-0.038,-0.017]	-0.009 [-0.024,0.005]
P(daily MME > 90)	0.041 [0.007,0.075]	-0.095 [-0.15,-0.041]	-0.082 [-0.15,-0.014]
P(4+4 within 90 days)	0 [-0.001,0]	-0.002 [-0.003,-0.001]	-0.001 [-0.001,0]
P(OUD and Overdose)	0.001 [-0.007,0.01]	0.017 [-0.008,0.043]	-0.008 [-0.025,0.009]
P(taking MAT drug)	-0.006 [-0.015,0.003]	0.015 [0.002,0.028]	0.013 [0.005,0.021]
	Prescription Limit	Good Samaritan	Naloxone Law
P(taking opioid)	0.062 [-0.257,0.381]	0.042 [-0.061,0.145]	0.163 [0.053,0.273]
P(overlapping claims)	-0.017 [-0.03,-0.004]	0.008 [-0.003,0.018]	-0.001 [-0.01,0.007]
P(daily MME > 90)	-0.127 [-0.229,-0.025]	-0.027 [-0.049,-0.005]	0.022 [-0.001,0.046]
P(4+4 within 90 days)	-0.002 [-0.003,-0.001]	0 [-0.001,0]	0 [-0.001,0]
P(OUD and Overdose)	0.021 [-0.004,0.047]	0.014 [0.002,0.027]	0.051 [0.03,0.073]
P(taking MAT drug)	0.034 [0.02,0.049]	0.007 [-0.003,0.016]	-0.001 [-0.007,0.005]

Note. We perform random-effects meta-analysis to summarize all temporal associations from t to t+12. The average effects with 95% confidence intervals are presented.

© 2021 Lee B et al. *JAMA Network Open.*

eTable 9. The Meta-Analysis of Quarterly Effects of State Policy Implementation on Overdose Deaths

	PDMP access	mandatory PDMP	Pill Mill Law
All overdose	-193.1 [-589.6,203.4]	-231.4 [-656.5,193.7]	-146.8 [-479,185.3]
Natural opioid	-183.9 [-345,-22.7]	-518.5 [-728.5,-308.5]	-182.9 [-434,68.3]
Methadone	-87.9 [-142.3,-33.5]	-122.7 [-207.5,-37.8]	-45.8 [-167.6,75.9]
Heroin	-40.7 [-228.7,147.3]	-115.9 [-298.6,66.8]	336.3 [79.5,593]
Synthetic opioid	380.3 [149.9,610.8]	12.2 [-350.8,375.3]	132.8 [-27,292.5]
Cocaine	103.7 [28,179.5]	-43.8 [-229,141.5]	97.3 [23.9,170.8]
	Prescription Limit	Good Samaritan	Naloxone Law
All overdose	3.4 [-625.8,632.7]	403.7 [172.7,634.8]	1344.3 [627.1,2061.6]
Natural opioid	13.6 [-232.2,259.5]	-64.6 [-178.4,49.2]	13.6 [-115.9,143.1]
Methadone	66.4 [-14.1,146.8]	-21.5 [-65.7,22.8]	-27.8 [-74.1,18.5]
Heroin	-146.5 [-441,147.9]	-9.6 [-118.8,99.6]	280.3 [50.7,509.9]
Synthetic opioid	-723.9 [-1419.7,-28.1]	58.3 [-90.4,207]	1338.2 [662.5,2014]
Cocaine	85.2 [-218.6,389]	-1.1 [-106.4,104.3]	557.6 [328.3,787]

Note. We perform random-effects meta-analysis to summarize all temporal associations from t to t+12. The average effects with 95% confidence intervals are presented.

© 2021 Lee B et al. *JAMA Network Open.*

eFigure 1. Illustration of Parallel Trends Assumption Under the Difference-in-Difference Framework: The Impact Of Mandatory PDMPs on All Overdose Deaths

Panel A shows how the parallel trends assumption allows difference-in-difference estimators to identify the causal effect of state policy treatment. Panel B shows quarterly trends of all overdose deaths before and after the implementation of mandatory PDMP across treated states (red), all unmatched controls (black), and matched controls based on panel matching (blue). Panel C shows marginal effects with 95% confidence intervals based on state-clustered standard errors from even study design, where we estimate the following models:$y_{st} = \sum_{l=-15}^{2} \alpha_l 1(T_{st} = l) + \sum_{f=0}^{15} \beta_f 1(T_{st} = f) + \theta_s + \gamma_t + \epsilon_{st}$, where T_{st} the number of quarters between quarter t and the policy implementation date in state s, and θ_s is a set of state fixed effects, and γ_t is a set of year-quarter fixed effects. Panel D shows marginal effects with 95% confidence intervals based on block-bootstrapped standard errors from panel matching.

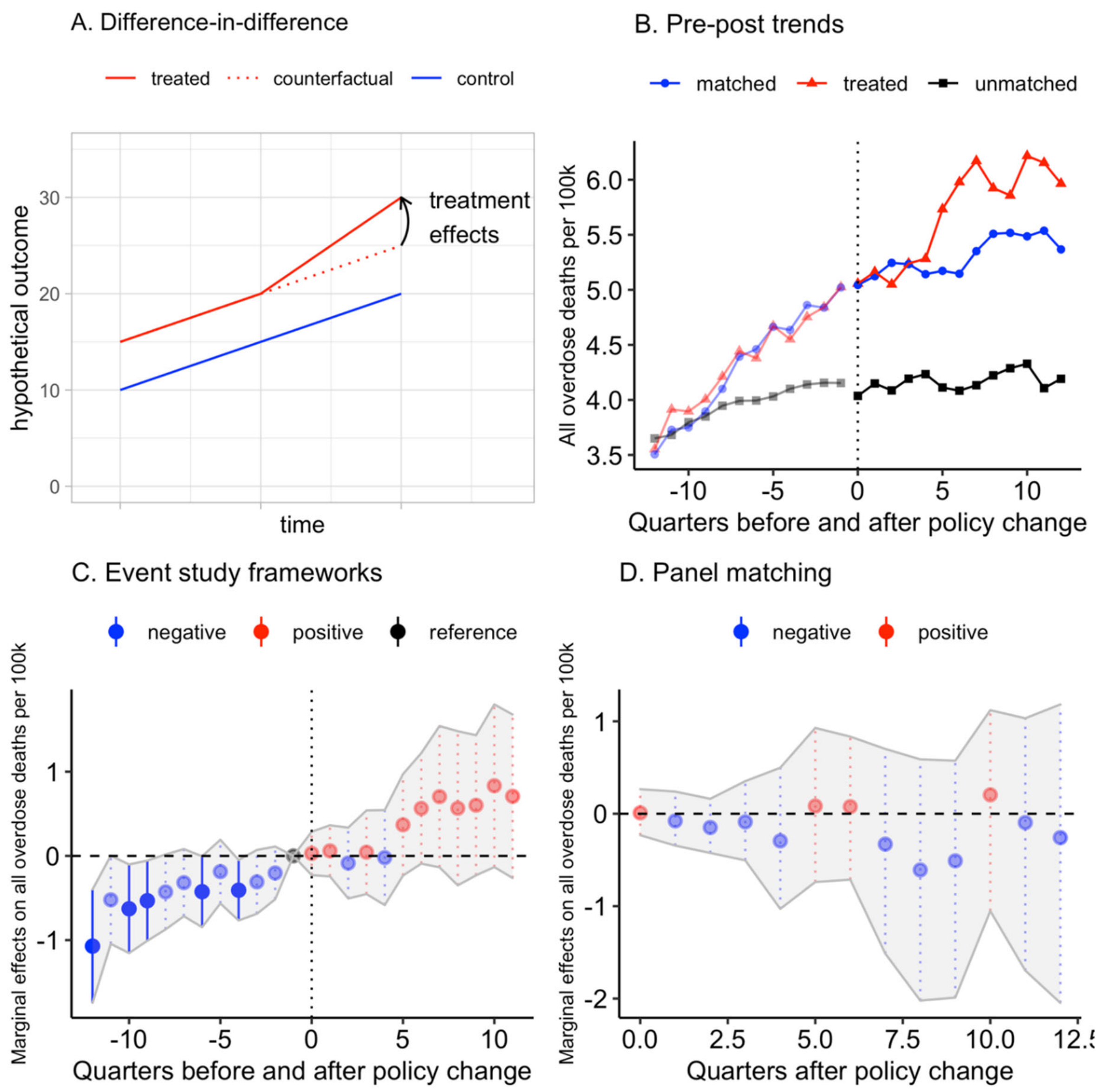

© 2021 Lee B et al. *JAMA Network Open.*

eFigure 2. Pre-Post Trends for Six Outcomes From a Commercially Insured Population Across Six Policies

Each panel shows quarterly trends for outcomes (on the column) before and after the implementation of each policy (on the row) across treated states (red), all unmatched controls (black), and matched controls based on panel matching (blue).

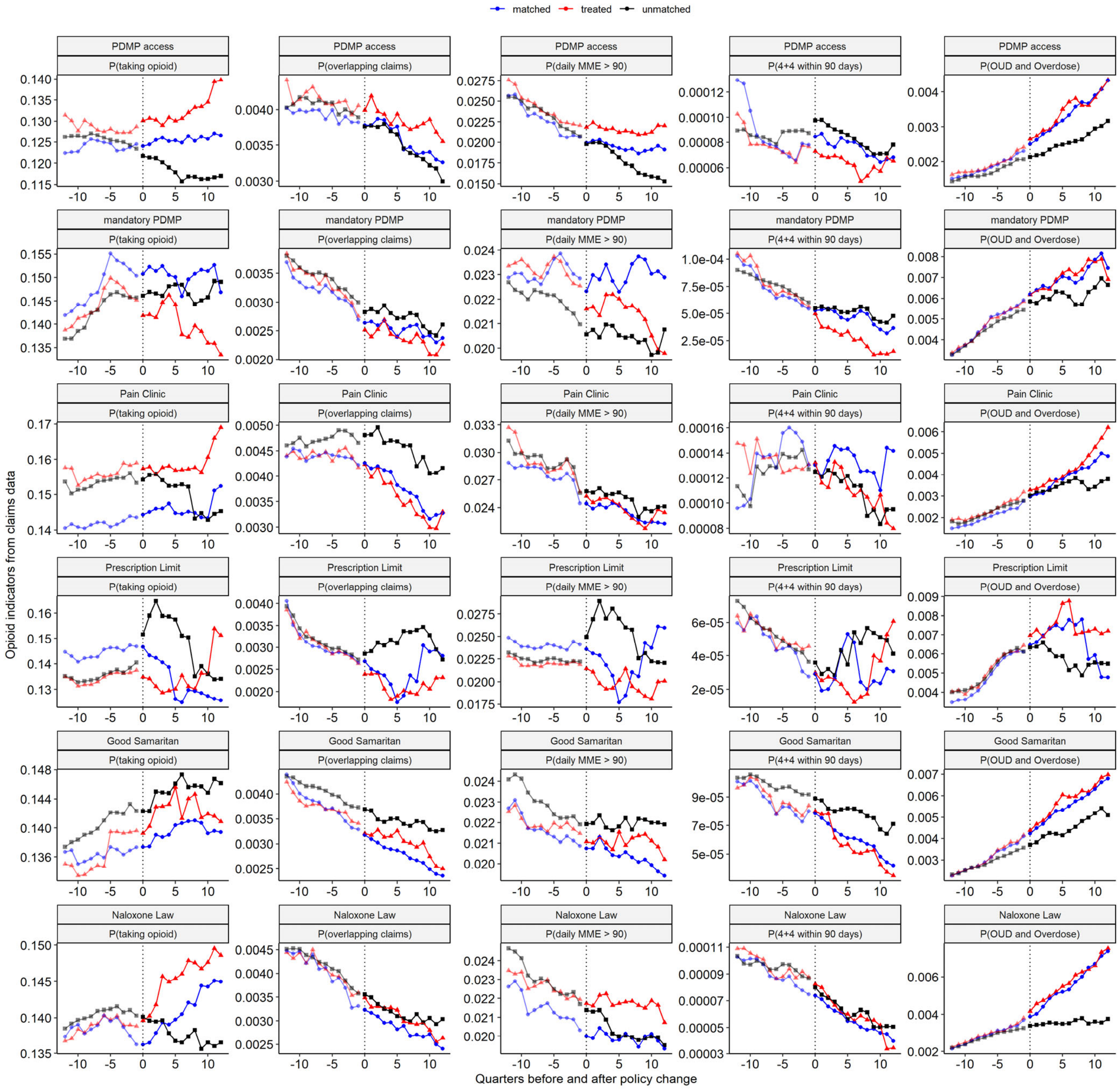

© 2021 Lee B et al. *JAMA Network Open.*

eFigure 3. Pre-Post Trends for Different Types of Drug-Related Deaths Across Six Policies

Each panel shows quarterly trends of outcomes (on the column) before and after the implementation of each policy (on the row) across treated states (red), all unmatched controls (black), and matched controls based on panel matching (blue).

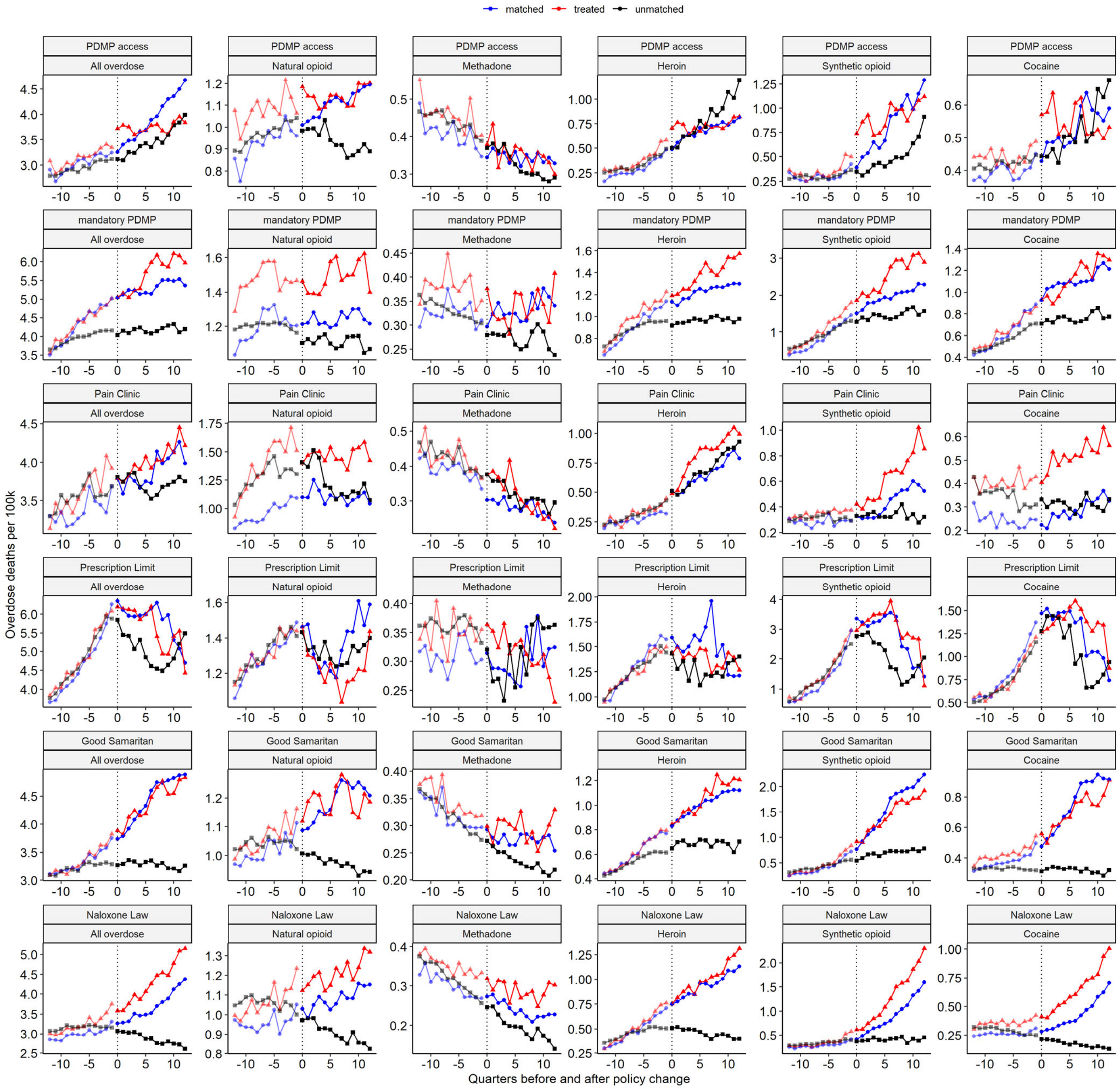

© 2021 Lee B et al. *JAMA Network Open.*

eFigure 4. Trends for Major Outcomes

The following eFigure4 depicts state-level opioid outcomes as a function of policy adoption. First, the rate of overdose deaths, as well as the diagnosis of overdose and opioid use disorder, have increased as states have implemented more state policies. Correspondingly, the use of opioid treatment has continuously increased as states have implemented more policies; the rate of Medication-Assisted Treatment (MAT) takers per 100,000 has more than quadrupled from 0.1% in a state without any policy to about 0.5% in a state with six policies. In contrast, indicators of opioid misuse and drug seeking have declined as policy implementation has increased. Namely, the proportion of patients taking a high-risk dosage and meeting the 4+4+90 threshold began to drop after the first and second adoption of state policies respectively, though the proportion of prescription opioid recipients exhibits some fluctuation.

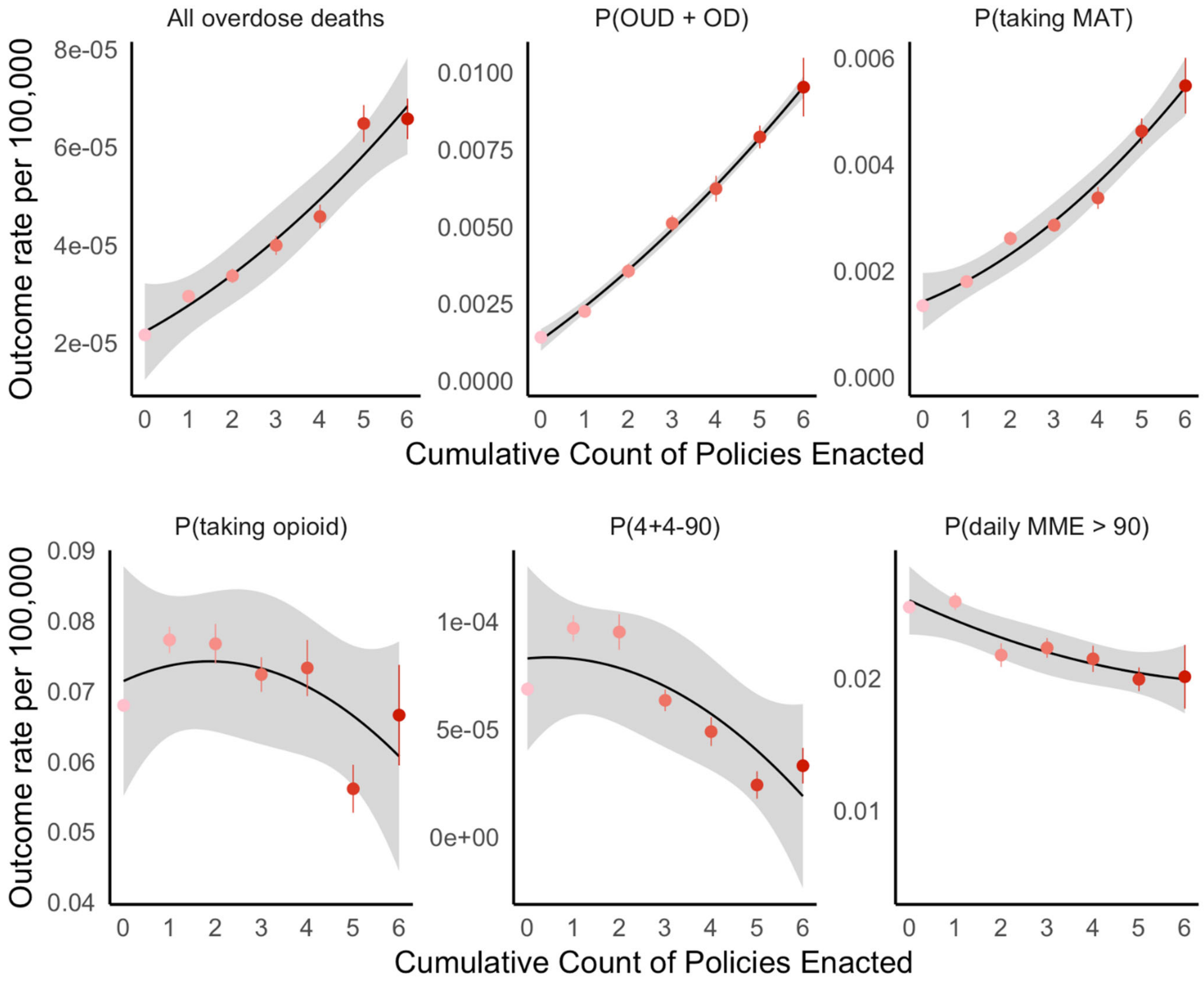

Note. Average mortality is calculated based on state-level data from the CDC Multiple Cause of Death database and all other measures are obtained from medical claims data from 29 million commercially-insured patients in the US from 2007 to 2018. All Opioid Mortality includes all overdose-related deaths. P(OUD + OD) indicates the proportion of patients with opioid use disorder and/or overdose. P

© 2021 Lee B et al. *JAMA Network Open.*

(taking opioid) indicates the proportion of prescription opioid takers. P(4+4+90) measures the proportion of opioid takers who visit 4+ unique doctors and 4+ unique pharmacies within 90 days. P(daily MME > 90) is calculated by the proportion of patients prescribed daily morphine mg equivalents (MME) greater than 90 mg. P(taking MAT) measures the proportion of patients prescribed any medication-assisted treatment (MAT) drug.

© 2021 Lee B et al. *JAMA Network Open.*

eFigure 5. The Association of State Policy Implementation With Indicators of Prescription Opioid Misuse and Opioid Overdose From a Commercially Insured Population

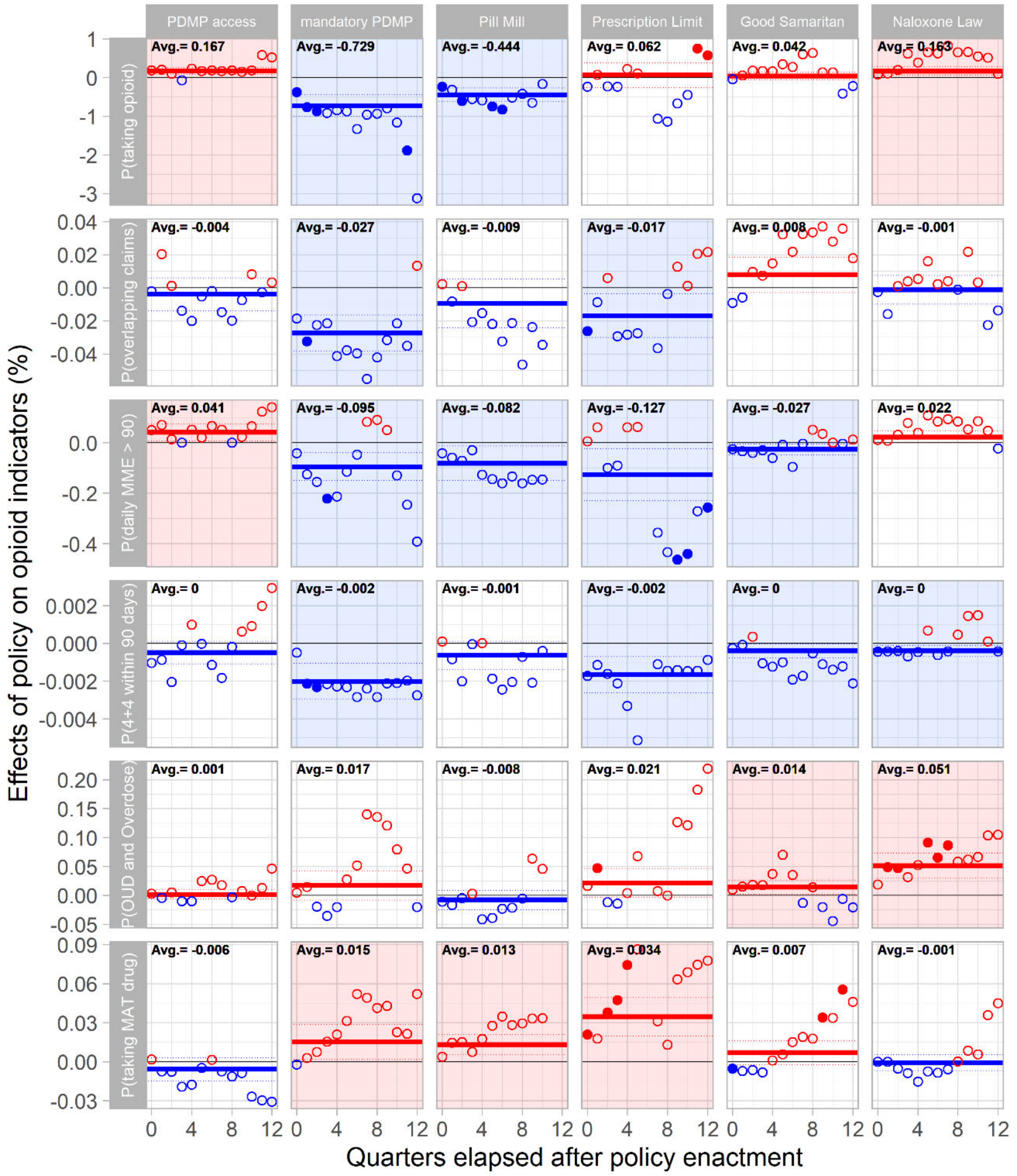

Note. Each dot represents the marginal effect of policy implementation on the future outcomes from 0 to 12 quarters after policy implementation from estimating panel matching models. The statistically significant effects ($p < 0.05$) are filled, otherwise empty. The horizontal lines show the average quarterly effects (noted in the upper-left corner) with 95% confidence intervals from meta-analysis using random-effects models across all over-time effects. When the overall effects across all quarters are significant, the background are colored by transparent red (positive effects) and blue (negative effects). See eTable 8 for meta-analysis results, and Appendix E for policy effects on all leading outcomes from t=0 to 12 quarters.

© 2021 Lee B et al. *JAMA Network Open.*

eFigure 6. The Association of State Policy Implementation With Different Types of Drug Overdose Deaths

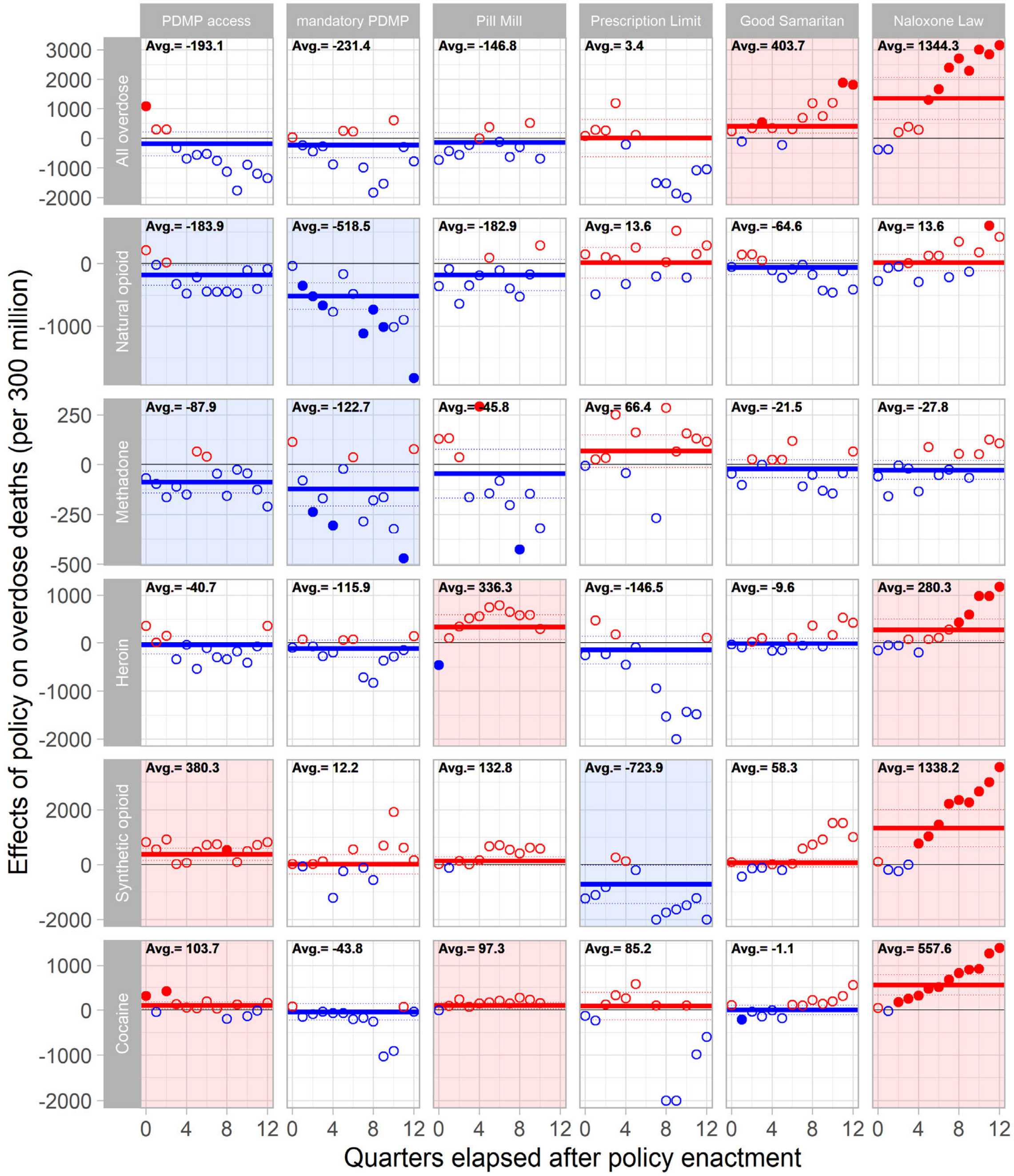

Note. Each dot represents the marginal effect of policy implementation on the future outcomes from 0 to 12 quarters after policy implementation from estimating panel matching models. The statistically significant effects ($p < 0.05$) are filled, otherwise empty. The horizontal lines show the average quarterly effects (noted in the upper-left corner) with 95% confidence intervals from meta-analysis using random-effects models across all over-time effects. When the overall effects across all quarters are significant, the background are colored by transparent red (positive effects) and blue (negative effects). See eTable 9 for meta-analysis results, and Appendix E for policy effects on all leading outcomes from t=0 to 12 quarters.

© 2021 Lee B et al. *JAMA Network Open.*

eTable 10. Regression Tables for Effects of State Policies on All Outcomes With 95% Confidence Intervals

time after policy change	Coef.	95% LCI	95% UCI	N treated	N total matched
Outcome P(taking opioid), Policy PDMP access					
t+0	5.6	-0.24	11	31	469
t+1	6.3	-0.31	13	30	448
t+2	3.1	-5	11	30	410
t+3	-1.9	-11	6.3	30	373
t+4	7	-3	16	30	355
t+5	5.2	-5.4	17	30	316
t+6	5.7	-4.6	16	30	289
t+7	5.1	-8	19	31	259
t+8	5.7	-7.3	20	30	236
t+9	4.7	-12	22	29	209
t+10	5.5	-11	23	29	194
t+11	17	-9.3	41	29	179
t+12	16	-10	40	29	152
time after policy change	Coef.	95% LCI	95% UCI	N treated	N total matched
Outcome P(taking opioid), Policy mandatory PDMP					
t+0	-11	-19	-3	32	569
t+1	-23	-40	-1.6	29	522
t+2	-26	-44	-2.9	28	480
t+3	-28	-51	3.8	24	442
t+4	-25	-54	7.9	24	410
t+5	-26	-64	11	22	362
t+6	-40	-95	9.6	19	321
t+7	-29	-74	6.5	16	289
t+8	-28	-77	7.7	15	266
t+9	-24	-67	13	15	236
t+10	-35	-81	2.6	15	209
t+11	-56	-110	-11	14	183
t+12	-93	-220	8.2	10	158
time after policy change	Coef.	95% LCI	95% UCI	N treated	N total matched
Outcome P(taking opioid), Policy Pain Clinic					
t+0	-7.1	-14	-0.36	10	52
t+1	-9.4	-20	0.73	10	50
t+2	-18	-31	-6.2	10	47
t+3	-16	-34	0.15	10	46
t+4	-18	-41	2.5	10	41
t+5	-22	-41	-6.7	10	36

© 2021 Lee B et al. *JAMA Network Open.*

t+6	-25	-50	-1.1	10	35
t+7	-15	-37	6	10	31
t+8	-13	-29	4.5	10	28
t+9	-19	-53	8.5	9	28
t+10	-4.8	-51	44	10	23
t+11					
t+12					
time after policy change	Coef.	95% LCI	95% UCI	N treated	N total matched
Outcome P(taking opioid), Policy Prescription Limit					
t+0	-7.1	-18	5.9	23	233
t+1	2.1	-15	18	23	173
t+2	-6.7	-27	16	18	156
t+3	-7	-29	18	17	171
t+4	6.6	-20	34	20	190
t+5	3.4	-27	37	14	167
t+6					
t+7	-32	-85	25	6	99
t+8	-34	-96	26	6	119
t+9	-20	-110	28	3	64
t+10	-13	-89	31	3	64
t+11	23	8.8	36	2	40
t+12	17	0.19	36	2	40
time after policy change	Coef.	95% LCI	95% UCI	N treated	N total matched
Outcome P(taking opioid), Policy Good Samaritan					
t+0	-1.1	-4.8	2.3	43	950
t+1	1.6	-2.7	6.5	43	889
t+2	5.4	-9.9	21	42	832
t+3	5.2	-11	23	42	771
t+4	4.7	-16	24	42	706
t+5	11	-12	36	39	655
t+6	8.2	-16	33	37	601
t+7	18	-4.7	40	36	541
t+8	19	-8.9	45	36	494
t+9	4.1	-31	41	34	422
t+10	4	-32	46	34	383
t+11	-12	-65	50	33	337
t+12	-6.6	-57	48	32	363
time after policy change	Coef.	95% LCI	95% UCI	N treated	N total matched
Outcome P(taking opioid), Policy Naloxone Law					
t+0	2.4	-3.3	7.3	45	1018

© 2021 Lee B et al. *JAMA Network Open.*

t+1	3.1	-1.6	8.1	44	914
t+2	5.8	-6.7	17	44	845
t+3	19	-5.4	38	45	764
t+4	12	-15	34	45	646
t+5	20	-9.5	46	45	567
t+6	19	-8.3	43	44	539
t+7	25	-5.8	53	44	483
t+8	20	-13	50	44	438
t+9	20	-11	50	41	468
t+10	16	-18	52	39	431
t+11	16	-17	47	38	438
t+12	3	-26	35	37	434
time after policy change	Coef.	95% LCI	95% UCI	N treated	N total matched
Outcome P(overlapping claims), Policy PDMP access					
t+0	-0.06	-0.72	0.58	31	469
t+1	0.61	-0.26	1.4	30	448
t+2	0.035	-0.83	1	30	410
t+3	-0.42	-1.3	0.48	30	373
t+4	-0.6	-1.4	0.12	30	355
t+5	-0.15	-1.4	1.1	30	316
t+6	-0.055	-1.5	1.5	30	289
t+7	-0.44	-1.6	0.87	31	259
t+8	-0.6	-2.1	0.98	30	236
t+9	-0.22	-1.9	1.3	29	209
t+10	0.25	-1.8	2	29	194
t+11	-0.081	-2	1.9	29	179
t+12	0.096	-2.4	2.3	29	152
time after policy change	Coef.	95% LCI	95% UCI	N treated	N total matched
Outcome P(overlapping claims), Policy mandatory PDMP					
t+0	-0.56	-1.1	0.053	32	569
t+1	-0.97	-1.6	-0.26	29	522
t+2	-0.68	-1.7	0.48	28	480
t+3	-0.65	-1.6	0.54	24	442
t+4	-1.2	-2.6	0.34	24	410
t+5	-1.1	-2.7	0.55	22	362
t+6	-1.2	-3	0.68	19	321
t+7	-1.7	-3.3	0.17	16	289
t+8	-1.3	-2.6	0.19	15	266
t+9	-0.95	-2.7	1.3	15	236
t+10	-0.64	-2.3	1.8	15	209

© 2021 Lee B et al. *JAMA Network Open.*

t+11	-1.1	-2.9	1.4	14	183
t+12	0.4	-2.7	4.9	10	158
time after policy change	Coef.	95% LCI	95% UCI	N treated	N total matched
Outcome P(overlapping claims), Policy Pain Clinic					
t+0	0.065	-0.78	1	10	52
t+1	-0.25	-1.1	0.75	10	50
t+2	0.029	-0.91	1.2	10	47
t+3	-0.62	-1.7	0.69	10	46
t+4	-0.46	-1.9	1.2	10	41
t+5	-0.66	-2.1	1.2	10	36
t+6	-0.97	-2.8	1.6	10	35
t+7	-0.64	-2.9	2.1	10	31
t+8	-1.4	-4.3	2.4	10	28
t+9	-0.71	-4.5	4	9	28
t+10	-1	-5	4.5	10	23
t+11					
t+12					
time after policy change	Coef.	95% LCI	95% UCI	N treated	N total matched
Outcome P(overlapping claims), Policy Prescription Limit					
t+0	-0.79	-1.5	-0.0038	23	233
t+1	-0.26	-0.95	0.5	23	173
t+2	0.18	-1.1	1.5	18	156
t+3	-0.88	-2	0.45	17	171
t+4	-0.85	-2	0.28	20	190
t+5	-0.82	-2	0.77	14	167
t+6					
t+7	-1.1	-3.2	4.2	6	99
t+8	-0.11	-3.2	6.3	6	119
t+9	0.38	-2.1	6.9	3	64
t+10	0.037	-2.2	6.5	3	64
t+11	0.62	-1.9	4.4	2	40
t+12	0.65	-1.8	3.9	2	40
time after policy change	Coef.	95% LCI	95% UCI	N treated	N total matched
Outcome P(overlapping claims), Policy Good Samaritan					
t+0	-0.27	-0.66	0.13	43	950
t+1	-0.17	-0.57	0.24	43	889
t+2	0.29	-0.41	0.98	42	832
t+3	0.22	-0.65	1	42	771
t+4	0.44	-0.66	1.6	42	706
t+5	0.97	-0.075	2.1	39	655

© 2021 Lee B et al. *JAMA Network Open.*

t+6	0.66	-0.58	1.9	37	601
t+7	0.98	-0.57	2.5	36	541
t+8	1	-0.71	2.6	36	494
t+9	1.1	-0.42	2.9	34	422
t+10	0.84	-1.1	3.1	34	383
t+11	1.1	-1.5	4	33	337
t+12	0.54	-2	3.2	32	363
time after policy change	Coef.	95% LCI	95% UCI	N treated	N total matched
Outcome P(overlapping claims), Policy Naloxone Law					
t+0	-0.076	-0.54	0.36	45	1018
t+1	-0.48	-1.1	0.17	44	914
t+2	0.03	-0.88	0.9	44	845
t+3	0.12	-0.87	1.1	45	764
t+4	0.16	-0.68	0.98	45	646
t+5	0.48	-0.52	1.4	45	567
t+6	0.069	-1.2	1.2	44	539
t+7	0.12	-1.1	1.3	44	483
t+8	-0.034	-1.6	1.3	44	438
t+9	0.65	-1.1	2.5	41	468
t+10	0.098	-1.9	2.2	39	431
t+11	-0.67	-2.6	1.2	38	438
t+12	-0.41	-2.1	1.5	37	434
time after policy change	Coef.	95% LCI	95% UCI	N treated	N total matched
Outcome P(daily MME > 90), Policy PDMP access					
t+0	1.5	-0.72	3.7	31	469
t+1	2.1	-0.82	4.8	30	448
t+2	0.41	-2	3.2	30	410
t+3	-0.0079	-2.6	2.7	30	373
t+4	1.5	-1.5	4.8	30	355
t+5	0.63	-3.8	4.6	30	316
t+6	2	-2.5	7	30	289
t+7	1.6	-3.5	6.6	31	259
t+8	-0.015	-5.4	5.3	30	236
t+9	0.71	-5	6.4	29	209
t+10	1.9	-4.1	8.1	29	194
t+11	3.7	-2.3	10	29	179
t+12	4.2	-3.3	11	29	152
time after policy change	Coef.	95% LCI	95% UCI	N treated	N total matched
Outcome P(daily MME > 90), Policy mandatory PDMP					
t+0	-1.3	-3.4	1.1	32	569

© 2021 Lee B et al. *JAMA Network Open.*

t+1	-3.8	-7.2	0.28	29	522
t+2	-4.6	-8.8	0.1	28	480
t+3	-6.6	-12	-0.72	24	442
t+4	-6.4	-13	2.4	24	410
t+5	-3.4	-11	5.2	22	362
t+6	-1.4	-7.9	5.8	19	321
t+7	2.5	-4.6	8.5	16	289
t+8	2.7	-6.7	11	15	266
t+9	1.5	-6.7	11	15	236
t+10	-3.9	-11	3.7	15	209
t+11	-7.4	-15	1.8	14	183
t+12	-12	-27	2.6	10	158
time after policy change	Coef.	95% LCI	95% UCI	N treated	N total matched
Outcome P(daily MME > 90), Policy Pain Clinic					
t+0	-1.3	-4.8	1.9	10	52
t+1	-1.8	-9.5	4.4	10	50
t+2	-2.1	-9.8	5	10	47
t+3	-0.89	-7.1	5.3	10	46
t+4	-3.8	-11	2.9	10	41
t+5	-4.3	-11	2.5	10	36
t+6	-4.8	-16	5.7	10	35
t+7	-4	-13	5.5	10	31
t+8	-4.8	-14	6.2	10	28
t+9	-4.4	-14	5.3	9	28
t+10	-4.4	-19	12	10	23
t+11					
t+12					
time after policy change	Coef.	95% LCI	95% UCI	N treated	N total matched
Outcome P(daily MME > 90), Policy Prescription Limit					
t+0	0.18	-2.6	3	23	233
t+1	1.8	-3.6	7.4	23	173
t+2	-3	-9.8	3.8	18	156
t+3	-2.7	-9.6	4.8	17	171
t+4	1.8	-5.6	10	20	190
t+5	1.9	-5.4	11	14	167
t+6					
t+7	-11	-19	0.23	6	99
t+8	-13	-24	1.9	6	119
t+9	-14	-34	-2.1	3	64
t+10	-13	-31	-0.88	3	64

© 2021 Lee B et al. *JAMA Network Open.*

t+11	-8.2	-15	0.75	2	40
t+12	-7.7	-15	-0.37	2	40
time after policy change	Coef.	95% LCI	95% UCI	N treated	N total matched
Outcome P(daily MME > 90), Policy Good Samaritan					
t+0	-0.77	-1.6	0.12	43	950
t+1	-0.99	-2.1	0.27	43	889
t+2	-1.2	-4.4	2.3	42	832
t+3	-0.87	-4.9	3.3	42	771
t+4	-1.8	-6.6	4	42	706
t+5	-0.22	-5.1	5.6	39	655
t+6	-2.9	-8.6	4.3	37	601
t+7	-0.11	-5.4	5.5	36	541
t+8	1.6	-3.5	6.6	36	494
t+9	1.1	-6	8.2	34	422
t+10	0.015	-7	8	34	383
t+11	-0.12	-9.1	9.8	33	337
t+12	0.38	-7.8	10	32	363
time after policy change	Coef.	95% LCI	95% UCI	N treated	N total matched
Outcome P(daily MME > 90), Policy Naloxone Law					
t+0	0.32	-0.75	1.2	45	1018
t+1	0.25	-1.1	1.7	44	914
t+2	0.95	-1.5	3.3	44	845
t+3	2.3	-1.9	6.8	45	764
t+4	1.2	-3.9	6.1	45	646
t+5	3.2	-1.3	7.5	45	567
t+6	2.5	-3	7.3	44	539
t+7	2.8	-3	8	44	483
t+8	2.5	-3.8	8.4	44	438
t+9	1.6	-3.5	6.2	41	468
t+10	2.6	-2.6	7.9	39	431
t+11	1.4	-4	6.7	38	438
t+12	-0.67	-5.3	4	37	434
time after policy change	Coef.	95% LCI	95% UCI	N treated	N total matched
Outcome P(4+4 within 90 days), Policy PDMP access					
t+0	-0.031	-0.067	0.0048	31	469
t+1	-0.026	-0.077	0.027	30	448
t+2	-0.062	-0.12	0.0031	30	410
t+3	-0.0031	-0.062	0.049	30	373
t+4	0.03	-0.058	0.11	30	355
t+5	-0.0009	-0.074	0.067	30	316

t+6	-0.034	-0.1	0.029	30	289
t+7	-0.055	-0.11	0.005	31	259
t+8	-0.0056	-0.077	0.063	30	236
t+9	0.019	-0.05	0.089	29	209
t+10	0.028	-0.047	0.11	29	194
t+11	0.06	-0.041	0.15	29	179
t+12	0.088	-0.038	0.21	29	152
time after policy change	Coef.	95% LCI	95% UCI	N treated	N total matched
Outcome P(4+4 within 90 days), Policy mandatory PDMP					
t+0	-0.015	-0.044	0.013	32	569
t+1	-0.064	-0.11	-0.015	29	522
t+2	-0.069	-0.12	-0.016	28	480
t+3	-0.065	-0.12	0.0067	24	442
t+4	-0.069	-0.14	0.02	24	410
t+5	-0.07	-0.14	0.026	22	362
t+6	-0.085	-0.17	0.015	19	321
t+7	-0.072	-0.15	0.02	16	289
t+8	-0.085	-0.17	0.016	15	266
t+9	-0.064	-0.17	0.07	15	236
t+10	-0.063	-0.18	0.072	15	209
t+11	-0.059	-0.18	0.1	14	183
t+12	-0.083	-0.18	0.084	10	158
time after policy change	Coef.	95% LCI	95% UCI	N treated	N total matched
Outcome P(4+4 within 90 days), Policy Pain Clinic					
t+0	0.0031	-0.019	0.03	10	52
t+1	-0.025	-0.069	0.026	10	50
t+2	-0.06	-0.12	0.0017	10	47
t+3	-0.00095	-0.055	0.063	10	46
t+4	0.00025	-0.057	0.065	10	41
t+5	-0.056	-0.14	0.042	10	36
t+6	-0.073	-0.18	0.061	10	35
t+7	-0.061	-0.18	0.084	10	31
t+8	-0.021	-0.12	0.088	10	28
t+9	-0.062	-0.18	0.075	9	28
t+10	-0.012	-0.14	0.14	10	23
t+11					
t+12					
time after policy change	Coef.	95% LCI	95% UCI	N treated	N total matched
Outcome P(4+4 within 90 days), Policy Prescription Limit					
t+0	-0.052	-0.099	0.0048	23	233

t+1	-0.035	-0.083	0.018	23	173
t+2	-0.048	-0.14	0.054	18	156
t+3	-0.063	-0.15	0.031	17	171
t+4	-0.099	-0.2	0.015	20	190
t+5	-0.15	-0.34	0.036	14	167
t+6					
t+7	-0.033	-0.14	0.21	6	99
t+8	-0.044	-0.15	0.21	6	119
t+9	-0.042	-0.15	0.17	3	64
t+10	-0.044	-0.13	0.16	3	64
t+11	-0.044	-0.19	0.15	2	40
t+12	-0.026	-0.23	0.22	2	40
time after policy change	Coef.	95% LCI	95% UCI	N treated	N total matched
Outcome P(4+4 within 90 days), Policy Good Samaritan					
t+0	-0.0078	-0.026	0.01	43	950
t+1	-0.0025	-0.023	0.016	43	889
t+2	0.011	-0.025	0.045	42	832
t+3	-0.031	-0.077	0.018	42	771
t+4	-0.037	-0.089	0.02	42	706
t+5	-0.03	-0.084	0.03	39	655
t+6	-0.058	-0.12	0.013	37	601
t+7	-0.052	-0.12	0.017	36	541
t+8	-0.016	-0.097	0.068	36	494
t+9	-0.033	-0.12	0.058	34	422
t+10	-0.042	-0.14	0.053	34	383
t+11	-0.037	-0.14	0.073	33	337
t+12	-0.064	-0.14	0.031	32	363
time after policy change	Coef.	95% LCI	95% UCI	N treated	N total matched
Outcome P(4+4 within 90 days), Policy Naloxone Law					
t+0	-0.013	-0.026	0.000082	45	1018
t+1	-0.013	-0.027	0.0015	44	914
t+2	-0.012	-0.049	0.03	44	845
t+3	-0.021	-0.079	0.032	45	764
t+4	-0.014	-0.1	0.059	45	646
t+5	0.021	-0.085	0.11	45	567
t+6	-0.018	-0.11	0.072	44	539
t+7	-0.013	-0.12	0.081	44	483
t+8	0.014	-0.081	0.1	44	438
t+9	0.044	-0.09	0.16	41	468
t+10	0.045	-0.11	0.19	39	431

© 2021 Lee B et al. *JAMA Network Open.*

t+11	0.0034	-0.14	0.14	38	438
t+12	-0.013	-0.14	0.11	37	434
time after policy change	Coef.	95% LCI	95% UCI	N treated	N total matched
Outcome P(Opioid Use Disorder and Overdose), Policy PDMP access					
t+0	0.096	-0.27	0.43	31	469
t+1	-0.13	-0.62	0.39	30	448
t+2	0.17	-0.55	0.92	30	410
t+3	-0.3	-1.2	0.67	30	373
t+4	-0.3	-1.4	1	30	355
t+5	0.75	-0.72	2.2	30	316
t+6	0.82	-1.1	2.7	30	289
t+7	0.53	-1.8	2.9	31	259
t+8	-0.088	-3.1	3	30	236
t+9	0.23	-3.2	3.8	29	209
t+10	0.0026	-4.3	4.3	29	194
t+11	0.39	-3.9	5.3	29	179
t+12	1.4	-2.7	5.8	29	152
time after policy change	Coef.	95% LCI	95% UCI	N treated	N total matched
Outcome P(Opioid Use Disorder and Overdose), Policy mandatory PDMP					
t+0	0.13	-1	1.3	32	569
t+1	0.43	-1.5	2.1	29	522
t+2	-0.59	-3.2	2	28	480
t+3	-1.1	-4.4	2.1	24	442
t+4	-0.6	-4.4	2.6	24	410
t+5	0.83	-3	4.1	22	362
t+6	1.6	-3.1	5.7	19	321
t+7	4.2	-0.29	7.2	16	289
t+8	4.1	-1	7.3	15	266
t+9	3.6	-3.1	7.9	15	236
t+10	2.4	-5.4	7.2	15	209
t+11	1.4	-9.6	8.9	14	183
t+12	-0.6	-14	5.7	10	158
time after policy change	Coef.	95% LCI	95% UCI	N treated	N total matched
Outcome P(Opioid Use Disorder and Overdose), Policy Pain Clinic					
t+0	-0.31	-1.2	0.46	10	52
t+1	-0.5	-1.9	0.84	10	50
t+2	-0.14	-1.6	1.1	10	47
t+3	0.089	-0.93	1.1	10	46
t+4	-1.2	-4.2	1.5	10	41
t+5	-1.2	-5.1	1.9	10	36

© 2021 Lee B et al. *JAMA Network Open.*

t+6	-0.69	-4.3	2.3	10	35
t+7	-0.64	-4.4	2.4	10	31
t+8	-0.16	-4	3.1	10	28
t+9	1.9	-3.1	5.7	9	28
t+10	1.4	-4.8	7.1	10	23
t+11					
t+12					
time after policy change	Coef.	95% LCI	95% UCI	N treated	N total matched
Outcome P(Opioid Use Disorder and Overdose), Policy Prescription Limit					
t+0	0.49	-0.71	1.7	23	233
t+1	1.4	0.019	2.8	23	173
t+2	-0.35	-2.4	1.6	18	156
t+3	-0.42	-4.1	2.5	17	171
t+4	0.12	-3.7	3.7	20	190
t+5	2	-2.6	5.5	14	167
t+6					
t+7	0.23	-10	4.2	6	99
t+8	0.0028	-11	5.1	6	119
t+9	3.8	-16	12	3	64
t+10	3.6	-17	13	3	64
t+11	5.5	-12	20	2	40
t+12	6.6	-9.3	19	2	40
time after policy change	Coef.	95% LCI	95% UCI	N treated	N total matched
Outcome P(Opioid Use Disorder and Overdose), Policy Good Samaritan					
t+0	0.29	-0.23	0.78	43	950
t+1	0.46	-0.38	1.3	43	889
t+2	0.54	-0.66	1.6	42	832
t+3	0.54	-1.1	1.8	42	771
t+4	1.1	-0.8	2.9	42	706
t+5	2.1	-0.39	4.4	39	655
t+6	1.1	-1.7	3.8	37	601
t+7	-0.39	-3.8	3.5	36	541
t+8	0.43	-3.5	4.4	36	494
t+9	-0.59	-5.5	4.4	34	422
t+10	-1.3	-7.6	5	34	383
t+11	-0.18	-6.2	5.8	33	337
t+12	-0.61	-7.5	5.6	32	363
time after policy change	Coef.	95% LCI	95% UCI	N treated	N total matched
Outcome P(Opioid Use Disorder and Overdose), Policy Naloxone Law					
t+0	0.56	-0.065	1.1	45	1018

© 2021 Lee B et al. *JAMA Network Open.*

t+1	1.5	0.5	2.3	44	914
t+2	1.4	0.13	2.6	44	845
t+3	0.95	-0.77	2.7	45	764
t+4	1.6	-0.26	3.5	45	646
t+5	2.7	0.82	4.6	45	567
t+6	2	0.085	4	44	539
t+7	2.6	0.21	5.2	44	483
t+8	1.7	-1.7	5.1	44	438
t+9	1.9	-1.4	5.2	41	468
t+10	2	-1.6	5.5	39	431
t+11	3.1	-0.78	7	38	438
t+12	3.1	-1.4	7.4	37	434
time after policy change	Coef.	95% LCI	95% UCI	N treated	N total matched
Outcome P(taking MAT drug), Policy PDMP access					
t+0	0.054	-0.41	0.45	31	469
t+1	-0.23	-0.95	0.49	30	448
t+2	-0.23	-0.87	0.41	30	410
t+3	-0.58	-1.7	0.47	30	373
t+4	-0.53	-1.7	0.74	30	355
t+5	-0.14	-1.2	0.93	30	316
t+6	0.041	-1.3	1.4	30	289
t+7	-0.22	-1.5	1.2	31	259
t+8	-0.34	-2.1	1.3	30	236
t+9	-0.26	-2.3	1.7	29	209
t+10	-0.8	-2.8	1.2	29	194
t+11	-0.89	-3.1	1.4	29	179
t+12	-0.92	-3	1.2	29	152
time after policy change	Coef.	95% LCI	95% UCI	N treated	N total matched
Outcome P(taking MAT drug), Policy mandatory PDMP					
t+0	-0.062	-0.46	0.35	32	569
t+1	0.084	-0.62	0.77	29	522
t+2	0.22	-0.72	1.3	28	480
t+3	0.46	-0.41	1.2	24	442
t+4	0.63	-0.69	1.9	24	410
t+5	0.94	-0.96	2.6	22	362
t+6	1.6	-0.71	3.5	19	321
t+7	1.5	-0.16	2.7	16	289
t+8	1.2	-0.72	2.5	15	266
t+9	1.3	-0.74	3.1	15	236
t+10	0.68	-2.3	3.1	15	209

t+11	0.65	-3.2	3.6	14	183
t+12	1.6	-2.1	4.3	10	158
time after policy change	Coef.	95% LCI	95% UCI	N treated	N total matched
Outcome P(taking MAT drug), Policy Pain Clinic					
t+0	0.12	-0.22	0.52	10	52
t+1	0.44	-0.017	0.85	10	50
t+2	0.45	-0.4	1.3	10	47
t+3	0.23	-0.76	1.2	10	46
t+4	0.53	-0.49	1.4	10	41
t+5	0.83	-0.41	2	10	36
t+6	1	-0.36	2.4	10	35
t+7	0.85	-1.1	2.4	10	31
t+8	0.89	-0.91	2.3	10	28
t+9	0.99	-0.99	2.7	9	28
t+10	1	-0.89	2.8	10	23
t+11					
t+12					
time after policy change	Coef.	95% LCI	95% UCI	N treated	N total matched
Outcome P(taking MAT drug), Policy Prescription Limit					
t+0	0.63	0.31	0.94	23	233
t+1	0.53	-0.26	1.3	23	173
t+2	1.1	0.042	2.1	18	156
t+3	1.4	0.12	2.5	17	171
t+4	2.2	0.56	3.6	20	190
t+5	2.6	-0.33	4.8	14	167
t+6					
t+7	0.94	-2.7	2.8	6	99
t+8	0.39	-4.3	3	6	119
t+9	1.9	-3.3	4.7	3	64
t+10	2.1	-4.4	4.9	3	64
t+11	2.2	-0.26	4.6	2	40
t+12	2.3	-0.59	5	2	40
time after policy change	Coef.	95% LCI	95% UCI	N treated	N total matched
Outcome P(taking MAT drug), Policy Good Samaritan					
t+0	-0.16	-0.29	-0.013	43	950
t+1	-0.21	-0.5	0.11	43	889
t+2	-0.19	-0.56	0.18	42	832
t+3	-0.25	-0.63	0.19	42	771
t+4	0.032	-0.54	0.6	42	706
t+5	0.17	-0.41	0.72	39	655

t+6	0.45	-0.35	1.3	37	601
t+7	0.57	-0.22	1.3	36	541
t+8	0.53	-0.53	1.5	36	494
t+9	1	0.049	2	34	422
t+10	1	-0.1	2.2	34	383
t+11	1.7	0.39	3	33	337
t+12	1.4	-0.28	3	32	363
time after policy change	Coef.	95% LCI	95% UCI	N treated	N total matched
Outcome P(taking MAT drug), Policy Naloxone Law					
t+0	-0.0036	-0.24	0.21	45	1018
t+1	-0.0033	-0.32	0.32	44	914
t+2	-0.16	-0.59	0.27	44	845
t+3	-0.26	-0.78	0.28	45	764
t+4	-0.46	-1.2	0.42	45	646
t+5	-0.22	-1.2	0.86	45	567
t+6	-0.25	-1.1	0.68	44	539
t+7	-0.18	-1.1	0.92	44	483
t+8	0.009	-0.99	1	44	438
t+9	0.26	-0.84	1.3	41	468
t+10	0.17	-1	1.3	39	431
t+11	1.1	-0.037	2.1	38	438
t+12	1.4	-0.069	2.6	37	434
time after policy change	Coef.	95% LCI	95% UCI	N treated	N total matched
Outcome All Opioid Mortality, Policy PDMP access					
t+0	36	1.8	68	31	469
t+1	9.8	-15	30	30	448
t+2	9.9	-19	38	30	410
t+3	-11	-34	12	30	373
t+4	-23	-47	6.5	30	355
t+5	-18	-50	17	30	316
t+6	-17	-58	24	30	289
t+7	-25	-75	30	31	259
t+8	-37	-89	24	31	237
t+9	-59	-130	29	30	211
t+10	-30	-120	67	29	194
t+11	-40	-140	74	29	179
t+12	-45	-160	74	29	152
time after policy change	Coef.	95% LCI	95% UCI	N treated	N total matched
Outcome All Opioid Mortality, Policy mandatory PDMP					
t+0	0.92	-23	27	33	571

t+1	-7.8	-34	24	32	528
t+2	-15	-42	16	29	482
t+3	-8.8	-50	35	28	450
t+4	-29	-100	50	24	410
t+5	8.3	-74	93	24	366
t+6	7.7	-71	83	22	327
t+7	-33	-150	70	19	295
t+8	-61	-200	59	16	268
t+9	-51	-200	57	15	236
t+10	20	-110	110	15	209
t+11	-9.5	-170	100	15	185
t+12	-26	-200	120	14	166
time after policy change	Coef.	95% LCI	95% UCI	N treated	N total matched
Outcome All Opioid Mortality, Policy Pain Clinic					
t+0	-25	-80	29	10	52
t+1	-14	-46	14	10	50
t+2	-18	-68	27	10	47
t+3	-7.4	-33	23	10	46
t+4	0.067	-38	36	10	41
t+5	13	-18	40	10	36
t+6	-4	-42	40	10	35
t+7	-21	-75	31	10	31
t+8	-9.7	-46	22	10	28
t+9	17	-24	57	9	28
t+10	-23	-73	25	10	23
t+11					
t+12					
time after policy change	Coef.	95% LCI	95% UCI	N treated	N total matched
Outcome All Opioid Mortality, Policy Prescription Limit					
t+0	2.5	-40	45	23	233
t+1	9.4	-42	64	23	173
t+2	8.9	-46	64	18	156
t+3	40	-38	130	17	171
t+4	-7.2	-70	54	20	190
t+5	3.8	-47	58	20	190
t+6					
t+7	-50	-140	28	12	171
t+8	-50	-310	93	6	119
t+9	-62	-370	91	6	118
t+10	-68	-440	100	3	64

t+11	-36	-380	120	3	61
t+12	-35	-240	89	2	40
time after policy change	Coef.	95% LCI	95% UCI	N treated	N total matched
Outcome All Opioid Mortality, Policy Good Samaritan					
t+0	7.9	-11	27	43	950
t+1	-3.7	-25	17	43	889
t+2	11	-6.6	30	43	842
t+3	18	0.18	37	42	771
t+4	11	-7.9	32	42	706
t+5	-7.5	-45	37	42	664
t+6	10	-31	56	39	613
t+7	23	-26	71	37	549
t+8	40	-10	91	36	494
t+9	25	-19	66	36	440
t+10	40	-16	87	34	383
t+11	63	9	120	34	348
t+12	61	6	110	33	375
time after policy change	Coef.	95% LCI	95% UCI	N treated	N total matched
Outcome All Opioid Mortality, Policy Naloxone Law					
t+0	-13	-28	5	45	1018
t+1	-12	-32	8.1	44	914
t+2	6.8	-16	31	44	845
t+3	13	-12	36	45	764
t+4	9.4	-11	31	45	646
t+5	43	18	72	45	567
t+6	56	27	85	45	541
t+7	80	37	120	44	483
t+8	90	44	140	44	438
t+9	76	33	120	44	477
t+10	100	53	150	41	443
t+11	95	42	150	39	446
t+12	110	50	170	38	443
time after policy change	Coef.	95% LCI	95% UCI	N treated	N total matched
Outcome Natural Opioid Mortality, Policy PDMP access					
t+0	7.1	-3.1	18	31	469
t+1	-0.56	-11	9	30	448
t+2	0.67	-12	13	30	410
t+3	-11	-27	5.9	30	373
t+4	-16	-31	2.7	30	355
t+5	-7.2	-23	11	30	316

© 2021 Lee B et al. *JAMA Network Open.*

t+6	-15	-31	3.5	30	289
t+7	-15	-34	5.9	31	259
t+8	-15	-29	0.85	31	237
t+9	-16	-36	5	30	211
t+10	-3.5	-25	18	29	194
t+11	-13	-47	16	29	179
t+12	-2.8	-27	21	29	152
time after policy change	Coef.	95% LCI	95% UCI	N treated	N total matched
Outcome Natural Opioid Mortality, Policy mandatory PDMP					
t+0	-1.2	-12	9.9	33	571
t+1	-12	-23	-0.95	32	528
t+2	-17	-32	-1.2	29	482
t+3	-22	-36	-9.3	28	450
t+4	-26	-49	0.43	24	410
t+5	-5.5	-24	12	24	366
t+6	-16	-39	6.2	22	327
t+7	-37	-71	-4.9	19	295
t+8	-24	-57	-0.24	16	268
t+9	-34	-65	-2.9	15	236
t+10	-34	-75	4.6	15	209
t+11	-30	-72	4.3	15	185
t+12	-61	-110	-21	14	166
time after policy change	Coef.	95% LCI	95% UCI	N treated	N total matched
Outcome Natural Opioid Mortality, Policy Pain Clinic					
t+0	-12	-36	10	10	52
t+1	-2.8	-26	23	10	50
t+2	-21	-58	15	10	47
t+3	-11	-34	15	10	46
t+4	-6.2	-33	25	10	41
t+5	3.1	-21	31	10	36
t+6	-3.6	-33	27	10	35
t+7	-13	-48	25	10	31
t+8	-18	-57	22	10	28
t+9	-5.6	-24	12	9	28
t+10	9.6	-20	45	10	23
t+11					
t+12					
time after policy change	Coef.	95% LCI	95% UCI	N treated	N total matched
Outcome Natural Opioid Mortality, Policy Prescription Limit					
t+0	4.9	-15	28	23	233

© 2021 Lee B et al. *JAMA Network Open.*

t+1	-16	-41	16	23	173
t+2	3.6	-13	19	18	156
t+3	2.1	-23	29	17	171
t+4	-11	-35	13	20	190
t+5	8.6	-16	36	20	190
t+6					
t+7	-6.9	-33	27	12	171
t+8	0.77	-45	41	6	119
t+9	17	-20	63	6	118
t+10	-7.3	-92	34	3	64
t+11	5.2	-65	41	3	61
t+12	9.6	-33	41	2	40
time after policy change	Coef.	95% LCI	95% UCI	N treated	N total matched
Outcome Natural Opioid Mortality, Policy Good Samaritan					
t+0	-1.7	-12	9.2	43	950
t+1	4.8	-5.2	14	43	889
t+2	4.9	-4.3	14	43	842
t+3	1.7	-9.2	12	42	771
t+4	-3.5	-16	11	42	706
t+5	-7.6	-24	11	42	664
t+6	-3	-17	13	39	613
t+7	-0.68	-13	13	37	549
t+8	-5.9	-20	9.4	36	494
t+9	-14	-29	0.57	36	440
t+10	-15	-32	0.76	34	383
t+11	-3.9	-23	14	34	348
t+12	-14	-34	6.8	33	375
time after policy change	Coef.	95% LCI	95% UCI	N treated	N total matched
Outcome Natural Opioid Mortality, Policy Naloxone Law					
t+0	-9.2	-19	1.1	45	1018
t+1	-2.3	-13	8	44	914
t+2	-1.5	-13	9.9	44	845
t+3	0.28	-11	11	45	764
t+4	-9.8	-26	7	45	646
t+5	4.3	-14	20	45	567
t+6	4.2	-6	16	45	541
t+7	-7.2	-19	5.7	44	483
t+8	12	-3.7	29	44	438
t+9	-4.2	-18	12	44	477
t+10	6	-12	25	41	443

© 2021 Lee B et al. *JAMA Network Open.*

t+11	20	2.3	40	39	446
t+12	14	-1.1	32	38	443
time after policy change	Coef.	95% LCI	95% UCI	N treated	N total matched
Outcome Methadone Mortality, Policy PDMP access					
t+0	-2.3	-11	6.2	31	469
t+1	-3.2	-8.5	2.2	30	448
t+2	-5.4	-11	0.82	30	410
t+3	-3.7	-8.9	1.7	30	373
t+4	-5	-9.9	0.096	30	355
t+5	2.2	-7.2	11	30	316
t+6	1.3	-5.2	7.6	30	289
t+7	-1.5	-7.3	3.8	31	259
t+8	-5.2	-10	0.93	31	237
t+9	-0.84	-6.6	4.8	30	211
t+10	-1.4	-11	7.2	29	194
t+11	-4.2	-12	3.8	29	179
t+12	-6.9	-18	3.2	29	152
time after policy change	Coef.	95% LCI	95% UCI	N treated	N total matched
Outcome Methadone Mortality, Policy mandatory PDMP					
t+0	3.8	-2.3	9.6	33	571
t+1	-2.6	-7.7	2	32	528
t+2	-7.9	-13	-1.9	29	482
t+3	-5.6	-11	0.089	28	450
t+4	-10	-18	-1.8	24	410
t+5	-0.72	-6.7	5.4	24	366
t+6	1.2	-7	9.9	22	327
t+7	-9.4	-21	4.7	19	295
t+8	-6	-16	4.3	16	268
t+9	-5.4	-16	4.1	15	236
t+10	-11	-25	0.0075	15	209
t+11	-16	-31	-3.1	15	185
t+12	2.6	-8.6	13	14	166
time after policy change	Coef.	95% LCI	95% UCI	N treated	N total matched
Outcome Methadone Mortality, Policy Pain Clinic					
t+0	4.3	-2.5	12	10	52
t+1	4.4	-2.5	11	10	50
t+2	1.2	-5.9	8.3	10	47
t+3	-5.4	-15	4.6	10	46
t+4	9.8	1.3	18	10	41
t+5	-4.8	-15	2.6	10	36

t+6	-2.7	-13	9.4	10	35
t+7	-6.7	-18	5	10	31
t+8	-14	-29	-1.9	10	28
t+9	-4.8	-16	4.5	9	28
t+10	-11	-22	0.25	10	23
t+11					
t+12					
time after policy change	Coef.	95% LCI	95% UCI	N treated	N total matched
Outcome Methadone Mortality, Policy Prescription Limit					
t+0	-0.23	-5.6	4.6	23	233
t+1	0.84	-7	9	23	173
t+2	1.1	-8.2	11	18	156
t+3	8.4	-0.15	18	17	171
t+4	-1.4	-13	9.8	20	190
t+5	5.4	-5	16	20	190
t+6					
t+7	-8.9	-26	8	12	171
t+8	9.5	-1.3	29	6	119
t+9	2.2	-6.6	13	6	118
t+10	5.2	-5	13	3	64
t+11	4.3	-6.7	19	3	61
t+12	3.8	-4.4	14	2	40
time after policy change	Coef.	95% LCI	95% UCI	N treated	N total matched
Outcome Methadone Mortality, Policy Good Samaritan					
t+0	-1.5	-5.1	2.4	43	950
t+1	-3.4	-7.1	0.15	43	889
t+2	0.86	-3.2	4.9	43	842
t+3	-0.061	-4.2	4.3	42	771
t+4	0.82	-4.3	5.7	42	706
t+5	0.82	-3.6	6.1	42	664
t+6	3.9	-0.15	8.2	39	613
t+7	-3.6	-9.1	2.3	37	549
t+8	-1.7	-7.3	4.1	36	494
t+9	-4.4	-11	2.4	36	440
t+10	-4.8	-11	1.8	34	383
t+11	-1.4	-7	4.6	34	348
t+12	2.2	-4.1	8.9	33	375
time after policy change	Coef.	95% LCI	95% UCI	N treated	N total matched
Outcome Methadone Mortality, Policy Naloxone Law					
t+0	-2	-5.7	1.7	45	1018

© 2021 Lee B et al. *JAMA Network Open.*

t+1	-5.3	-10	0.18	44	914
t+2	-0.17	-4	3.7	44	845
t+3	-0.66	-5.6	4.8	45	764
t+4	-4.5	-9.8	0.81	45	646
t+5	2.9	-3	8.4	45	567
t+6	-1.7	-8	4.6	45	541
t+7	-0.84	-6.4	5.5	44	483
t+8	1.8	-5.9	8.3	44	438
t+9	-2.2	-8.1	3.9	44	477
t+10	1.7	-6.2	9.9	41	443
t+11	4.2	-3.6	12	39	446
t+12	3.5	-5	12	38	443
time after policy change	Coef.	95% LCI	95% UCI	N treated	N total matched
Outcome Heroin Mortality, Policy PDMP access					
t+0	12	-9.8	30	31	469
t+1	0.55	-12	13	30	448
t+2	5.2	-12	22	30	410
t+3	-11	-29	6.7	30	373
t+4	-1	-16	15	30	355
t+5	-18	-50	19	30	316
t+6	-3.6	-29	26	30	289
t+7	-9.8	-33	19	31	259
t+8	-11	-42	25	31	237
t+9	-5.8	-38	30	30	211
t+10	-14	-60	36	29	194
t+11	-2.2	-44	41	29	179
t+12	12	-15	41	29	152
time after policy change	Coef.	95% LCI	95% UCI	N treated	N total matched
Outcome Heroin Mortality, Policy mandatory PDMP					
t+0	-3.3	-15	8.5	33	571
t+1	2.7	-12	18	32	528
t+2	-2.4	-16	13	29	482
t+3	-9.2	-26	8.4	28	450
t+4	-6.6	-33	19	24	410
t+5	2.3	-25	30	24	366
t+6	2.7	-29	31	22	327
t+7	-24	-59	6.9	19	295
t+8	-28	-74	12	16	268
t+9	-12	-65	32	15	236
t+10	-9.4	-72	37	15	209

© 2021 Lee B et al. *JAMA Network Open.*

t+11	-4.9	-63	43	15	185
t+12	5.1	-47	54	14	166
time after policy change	Coef.	95% LCI	95% UCI	N treated	N total matched
Outcome Heroin Mortality, Policy Pain Clinic					
t+0	-15	-32	-0.3	10	52
t+1	3.7	-5.6	11	10	50
t+2	12	-2.4	23	10	47
t+3	17	-3.1	34	10	46
t+4	19	-4.5	37	10	41
t+5	25	-7.2	52	10	36
t+6	26	-0.58	50	10	35
t+7	22	-13	48	10	31
t+8	20	-21	54	10	28
t+9	20	-36	61	9	28
t+10	10	-74	71	10	23
t+11					
t+12					
time after policy change	Coef.	95% LCI	95% UCI	N treated	N total matched
Outcome Heroin Mortality, Policy Prescription Limit					
t+0	-8.4	-30	13	23	233
t+1	16	-11	40	23	173
t+2	-7.6	-25	13	18	156
t+3	6.4	-22	31	17	171
t+4	-15	-55	21	20	190
t+5	-2.9	-45	33	20	190
t+6					
t+7	-31	-80	16	12	171
t+8	-51	-150	14	6	119
t+9	-74	-210	7.7	6	118
t+10	-48	-280	60	3	64
t+11	-49	-300	49	3	61
t+12	3.9	-96	70	2	40
time after policy change	Coef.	95% LCI	95% UCI	N treated	N total matched
Outcome Heroin Mortality, Policy Good Samaritan					
t+0	-0.82	-8.3	6.3	43	950
t+1	-3	-9.7	3.1	43	889
t+2	1.1	-7.7	9.4	43	842
t+3	3.6	-9	18	42	771
t+4	-5.4	-20	9.7	42	706
t+5	-4.8	-21	14	42	664

t+6	3.8	-20	28	39	613
t+7	-1.5	-26	27	37	549
t+8	12	-15	40	36	494
t+9	-2.2	-33	32	36	440
t+10	5.8	-27	39	34	383
t+11	18	-9.3	44	34	348
t+12	14	-14	41	33	375
time after policy change	Coef.	95% LCI	95% UCI	N treated	N total matched
Outcome Heroin Mortality, Policy Naloxone Law					
t+0	-5.1	-12	2.7	45	1018
t+1	-1.3	-8.1	4.8	44	914
t+2	-1.6	-9.3	6.5	44	845
t+3	2.8	-8.2	14	45	764
t+4	-6.6	-17	3.9	45	646
t+5	2.8	-9.4	15	45	567
t+6	4	-7.4	16	45	541
t+7	9.6	-2	20	44	483
t+8	15	2.7	26	44	438
t+9	20	7.5	34	44	477
t+10	33	14	51	41	443
t+11	33	16	48	39	446
t+12	39	17	59	38	443
time after policy change	Coef.	95% LCI	95% UCI	N treated	N total matched
Outcome Synthetic Mortality, Policy PDMP access					
t+0	28	-5.7	54	31	469
t+1	19	-4.6	38	30	448
t+2	31	-0.43	55	30	410
t+3	0.94	-9.3	10	30	373
t+4	2.4	-6.7	11	30	355
t+5	16	-2.2	31	30	316
t+6	25	-14	54	30	289
t+7	25	-6.3	57	31	259
t+8	18	1.9	35	31	237
t+9	3.2	-30	38	30	211
t+10	17	-47	77	29	194
t+11	25	-41	85	29	179
t+12	28	-31	85	29	152
time after policy change	Coef.	95% LCI	95% UCI	N treated	N total matched
Outcome Synthetic Mortality, Policy mandatory PDMP					
t+0	0.69	-19	20	33	571

© 2021 Lee B et al. *JAMA Network Open.*

t+1	-1.9	-26	26	32	528
t+2	0.84	-26	30	29	482
t+3	4.3	-27	37	28	450
t+4	-40	-110	25	24	410
t+5	-7.7	-81	60	24	366
t+6	19	-57	87	22	327
t+7	-4	-120	100	19	295
t+8	-19	-190	120	16	268
t+9	23	-130	130	15	236
t+10	64	-96	180	15	209
t+11	21	-140	140	15	185
t+12	5.8	-200	170	14	166
time after policy change	Coef.	95% LCI	95% UCI	N treated	N total matched
Outcome Synthetic Mortality, Policy Pain Clinic					
t+0	0.7	-12	12	10	52
t+1	-3.6	-12	2.6	10	50
t+2	4.7	-6.6	14	10	47
t+3	0.52	-10	9.8	10	46
t+4	5.7	-8.8	17	10	41
t+5	23	-6.6	44	10	36
t+6	24	-13	55	10	35
t+7	19	-9.5	40	10	31
t+8	14	-21	42	10	28
t+9	21	-6.2	45	9	28
t+10	20	-28	55	10	23
t+11					
t+12					
time after policy change	Coef.	95% LCI	95% UCI	N treated	N total matched
Outcome Synthetic Mortality, Policy Prescription Limit					
t+0	-41	-80	2.6	23	233
t+1	-37	-99	27	23	173
t+2	-27	-96	36	18	156
t+3	9.2	-75	92	17	171
t+4	4.5	-61	58	20	190
t+5	-6.8	-62	41	20	190
t+6					
t+7	-81	-240	33	12	171
t+8	-58	-390	99	6	119
t+9	-54	-400	110	6	118
t+10	-49	-420	92	3	64

© 2021 Lee B et al. *JAMA Network Open.*

t+11	-41	-350	82	3	61
t+12	-130	-390	59	2	40
time after policy change	Coef.	95% LCI	95% UCI	N treated	N total matched
Outcome Synthetic Mortality, Policy Good Samaritan					
t+0	3.4	-2.5	10	43	950
t+1	-15	-30	4.2	43	889
t+2	-4.8	-19	10	43	842
t+3	-3.6	-22	17	42	771
t+4	0.41	-18	20	42	706
t+5	-6.7	-42	31	42	664
t+6	1.2	-44	43	39	613
t+7	20	-34	75	37	549
t+8	25	-33	78	36	494
t+9	31	-18	77	36	440
t+10	51	-7.5	99	34	383
t+11	51	-7.4	96	34	348
t+12	34	-41	100	33	375
time after policy change	Coef.	95% LCI	95% UCI	N treated	N total matched
Outcome Synthetic Mortality, Policy Naloxone Law					
t+0	3.6	-5	12	45	1018
t+1	-6.2	-19	8.5	44	914
t+2	-7.9	-31	22	44	845
t+3	-0.16	-21	24	45	764
t+4	26	14	37	45	646
t+5	35	16	51	45	567
t+6	49	26	70	45	541
t+7	74	42	100	44	483
t+8	79	43	110	44	438
t+9	76	42	110	44	477
t+10	89	36	140	41	443
t+11	100	56	150	39	446
t+12	120	63	170	38	443
time after policy change	Coef.	95% LCI	95% UCI	N treated	N total matched
Outcome Cocaine Mortality, Policy PDMP access					
t+0	10	0.36	19	31	469
t+1	-1.4	-10	7.7	30	448
t+2	14	0.021	26	30	410
t+3	4.4	-1.3	9.6	30	373
t+4	1.8	-4.4	7.4	30	355
t+5	1.3	-7.8	11	30	316

© 2021 Lee B et al. *JAMA Network Open.*

t+6	6.4	-1.2	14	30	289
t+7	1	-11	14	31	259
t+8	-6.5	-19	7.1	31	237
t+9	4.1	-4.3	13	30	211
t+10	-4.4	-23	17	29	194
t+11	-0.32	-14	13	29	179
t+12	5.4	-8	19	29	152
time after policy change	Coef.	95% LCI	95% UCI	N treated	N total matched
Outcome Cocaine Mortality, Policy mandatory PDMP					
t+0	2.6	-6.8	13	33	571
t+1	-5.1	-19	12	32	528
t+2	-2.9	-15	9.6	29	482
t+3	-1.3	-18	15	28	450
t+4	-2.3	-29	21	24	410
t+5	-2.1	-34	28	24	366
t+6	-6.8	-40	22	22	327
t+7	-5.7	-55	32	19	295
t+8	-8.5	-64	36	16	268
t+9	-34	-130	36	15	236
t+10	-30	-150	60	15	209
t+11	2.3	-72	58	15	185
t+12	-1.2	-69	55	14	166
time after policy change	Coef.	95% LCI	95% UCI	N treated	N total matched
Outcome Cocaine Mortality, Policy Pain Clinic					
t+0	-0.2	-3.8	3.9	10	52
t+1	3.2	-2.8	8.9	10	50
t+2	8	-5.5	20	10	47
t+3	2.4	-3.6	8.6	10	46
t+4	4.8	-6	14	10	41
t+5	5.6	-6.8	16	10	36
t+6	6.8	-5.7	18	10	35
t+7	5	-6.7	16	10	31
t+8	9.3	-4.5	20	10	28
t+9	7.6	-5.8	20	9	28
t+10	5.3	-3.5	13	10	23
t+11					
t+12					
time after policy change	Coef.	95% LCI	95% UCI	N treated	N total matched
Outcome Cocaine Mortality, Policy Prescription Limit					
t+0	-4.2	-24	18	23	233

© 2021 Lee B et al. *JAMA Network Open.*

t+1	-7.8	-33	19	23	173
t+2	4.2	-23	26	18	156
t+3	11	-21	37	17	171
t+4	8.6	-17	33	20	190
t+5	19	-12	50	20	190
t+6					
t+7	3.3	-52	50	12	171
t+8	-140	-520	49	6	119
t+9	-87	-330	35	6	118
t+10	3.3	-120	63	3	64
t+11	-33	-170	23	3	61
t+12	-20	-98	28	2	40
time after policy change	Coef.	95% LCI	95% UCI	N treated	N total matched
Outcome Cocaine Mortality, Policy Good Samaritan					
t+0	3.6	-1.1	8.1	43	950
t+1	-6.9	-12	-2.1	43	889
t+2	-1.1	-6	4.5	43	842
t+3	-4.8	-12	3.3	42	771
t+4	-0.29	-9.4	9.4	42	706
t+5	-6	-20	11	42	664
t+6	4	-9.7	20	39	613
t+7	3.4	-11	20	37	549
t+8	7.4	-11	25	36	494
t+9	4.6	-13	24	36	440
t+10	6.3	-15	24	34	383
t+11	10	-12	28	34	348
t+12	19	-5.4	41	33	375
time after policy change	Coef.	95% LCI	95% UCI	N treated	N total matched
Outcome Cocaine Mortality, Policy Naloxone Law					
t+0	1.5	-3.8	6.6	45	1018
t+1	-0.68	-6.1	4.6	44	914
t+2	5.9	0.07	12	44	845
t+3	8.4	3.5	13	45	764
t+4	11	4.9	17	45	646
t+5	16	11	21	45	567
t+6	17	10	24	45	541
t+7	23	14	30	44	483
t+8	27	18	37	44	438
t+9	30	18	43	44	477
t+10	31	19	42	41	443

t+11	42	26	57	39	446
t+12	46	30	62	38	443

© 2021 Lee B et al. *JAMA Network Open.*

eReferences

1 Kiang MV, Humphreys K, Cullen MR, Basu S. Opioid prescribing patterns among medical providers in the United States, 2003-17: retrospective, observational study. *BMJ* 2020; **368**. DOI:10.1136/bmj.l6968.

2 Perry BL, Yang KC, Kaminski P, *et al.* Co-prescription network reveals social dynamics of opioid doctor shopping. *PLOS ONE* 2019; **14**: e0223849.

3 Wallace PJ, Shah ND, Dennen T, Bleicher PA, Crown WH. Optum Labs: Building A Novel Node In The Learning Health Care System. *Health Aff (Millwood)* 2014; **33**: 1187–94.

4 Jeffery MM, Hooten WM, Henk HJ, *et al.* Trends in opioid use in commercially insured and Medicare Advantage populations in 2007-16: retrospective cohort study. *BMJ* 2018; **362**. DOI:10.1136/bmj.k2833.

5 Horwitz J, Davis C, McClelland L, Fordon R, Meara E. The Problem of Data Quality in Analyses of Opioid Regulation: The Case of Prescription Drug Monitoring Programs. Cambridge, MA: National Bureau of Economic Research, 2018.

6 Davis CS, Lieberman AJ, Hernandez-Delgado H, Suba C. Laws limiting the prescribing or dispensing of opioids for acute pain in the United States: A national systematic legal review. *Drug Alcohol Depend* 2019; **194**: 166–72.

7 Imai K, Kim IS, Wang E. Matching Methods for Causal Inference with Time-Series Cross-Sectional Data. ; : 37.

8 Ho D, Imai K, King G, Stuart EA. Matching as Nonparametric Preprocessing for Reducing Model Dependence in Parametric Causal Inference. *Polit Anal* 2007; **15**: 199–236.

9 Imai K, Ratkovic M. Covariate balancing propensity score. *J R Stat Soc Ser B Stat Methodol* 2014; **76**: 243–63.

10 Wen H, Soni A, Hollingsworth A, *et al.* Association Between Medicaid Expansion and Rates of Opioid-Related Hospital Use. *JAMA Intern Med* 2020; published online March 23. DOI:10.1001/jamainternmed.2020.0473.

11 Goodman-Bacon A. Difference-in-Differences with Variation in Treatment Timing. National Bureau of Economic Research, 2018 DOI:10.3386/w25018.

12 Imai K, Kim IS. On the Use of Two-way Fixed Effects Regression Models for Causal Inference with Panel Data. https://imai.fas.harvard.edu/research/twoway.html.

13 Bertrand M, Duflo E, Mullainathan S. How Much Should We Trust Differences-In-Differences Estimates? *Q J Econ* 2004; **119**: 249–75.

© 2021 Lee B et al. *JAMA Network Open.*

14 Abraham S, Sun L. Estimating Dynamic Treatment Effects in Event Studies with Heterogeneous Treatment Effects. *ArXiv180405785 Econ* 2018; published online April 16. http://arxiv.org/abs/1804.05785 (accessed March 4, 2019).

15 Borusyak K, Jaravel X. Revisiting Event Study Designs. *SSRN Electron J* 2016. DOI:10.2139/ssrn.2826228.

16 Viechtbauer W. Conducting meta-analyses in R with the metafor package. *J Stat Softw* 2010; **36**: 1–48.

17 Pauly NJ, Slavova S, Delcher C, Freeman PR, Talbert J. Features of prescription drug monitoring programs associated with reduced rates of prescription opioid-related poisonings. *Drug Alcohol Depend* 2018; **184**: 26–32.

18 PEW. Prescription Drug Monitoring Programs: Evidence-based practices to optimize prescriber use. 2016; published online Dec. https://www.pewtrusts.org/en/research-and-analysis/reports/2016/12/prescription-drug-monitoring-programs.

19 Buchmueller TC, Carey C. The Effect of Prescription Drug Monitoring Programs on Opioid Utilization in Medicare. *Am Econ J Econ Policy* 2018; **10**: 77–112.

20 Sacks DW, Hollingsworth A, Nguyen TD, Simon KI. Can Policy Affect Initiation of Addictive Substance Use? Evidence from Opioid Prescribing. National Bureau of Economic Research, 2019 DOI:10.3386/w25974.

21 Mallatt J. The Effect of Prescription Drug Monitoring Programs on Opioid Prescriptions and Heroin Crime Rates. Rochester, NY: Social Science Research Network, 2018 DOI:10.2139/ssrn.3050692.

22 Meinhofer A. Prescription Drug Monitoring Programs: The Role of Asymmetric Information on Drug Availability and Abuse. *Am J Health Econ* 2018; **4**: 504–26.

© 2021 Lee B et al. *JAMA Network Open.*
